# Quorum Sensing and Antimicrobial Production Orchestrate Biofilm Dynamics in Multispecies Bacterial Communities

**DOI:** 10.1128/spectrum.02615-22

**Published:** 2022-10-18

**Authors:** April C. Armes, Jillian L. Walton, Alison Buchan

**Affiliations:** a Department of Microbiology, University of Tennessee, Knoxville, Tennessee, USA; University of Massachusetts Amherst

**Keywords:** microbial interactions, AHL, quorum sensing, biofilms, secondary metabolites, *Roseobacteraceae*, roseobacters

## Abstract

Microbial interactions are often mediated by diffusible small molecules, including secondary metabolites, that play roles in cell-to-cell signaling and inhibition of competitors. Biofilms are often “hot spots” for high concentrations of bacteria and their secondary metabolites, which make them ideal systems for the study of small-molecule contributions to microbial interactions. Here, we use a five-member synthetic community consisting of *Roseobacteraceae* representatives to investigate the role of secondary metabolites on microbial biofilm dynamics. One synthetic community member, *Rhodobacterales* strain Y4I, possesses two acylated homoserine lactone (AHL)-based cell-to-cell signaling systems (*pgaRI* and *phaRI*) as well as a nonribosomal peptide synthase gene (*igi*) cluster that encodes the antimicrobial indigoidine. Through serial substitution of Y4I with mutants deficient in single signaling molecule pathways, the contribution of these small-molecule systems could be assessed. As secondary metabolite production is dependent upon central metabolites, the influence of growth substrate (i.e., complex medium versus defined medium with a single carbon substrate) on these dynamics was also considered. Depending on the Y4I mutant genotype included, community dynamics ranged from competitive to cooperative. The observed interactions were mostly competitive in nature. However, the community harboring a Y4I variant that was both impaired in quorum sensing (QS) pathways and unable to produce indigoidine (*pgaR* variant) shifted toward more cooperative interactions over time. These cooperative interactions were enhanced in the defined growth medium. The results presented provide a framework for deciphering complex, small-molecule-mediated interactions that have broad application to microbial biology.

**IMPORTANCE** Microbial biofilms play critical roles in marine ecosystems and are hot spots for microbial interactions that play a role in the development and function of these communities. *Roseobacteraceae* are an abundant and active family of marine heterotrophic bacteria forming close associations with phytoplankton and carrying out key transformations in biogeochemical cycles. Group members are aggressive primary colonizers of surfaces, where they set the stage for the development of multispecies biofilm communities. Few studies have examined the impact of secondary metabolites, such as cell-to-cell signaling and antimicrobial production, on marine microbial biofilm community structure. Here, we assessed the impact of secondary metabolites on microbial interactions using a synthetic, five-member *Roseobacteraceae* community by measuring species composition and biomass production during biofilm growth. We present evidence that secondary metabolites influence social behaviors within these multispecies microbial biofilms, thereby improving understanding of bacterial secondary metabolite production influence on social behaviors within marine microbial biofilm communities.

## INTRODUCTION

Microbial interactions play critical roles in defining microbial community structure and function. These microbial interactions span from cooperative interactions, such as resistance to antimicrobials and cometabolism (1–3), to more competitive interactions, including the production of inhibitory compounds and resource acquisition (4–6). Many types of microbial interactions are mediated by small molecules. While not essential to primary metabolism, these small molecules (i.e., secondary metabolites) facilitate interactions between microbes and their biotic and abiotic environments (7). Two common classes of secondary metabolites produced by microbes are signaling molecules and antimicrobial compounds. A well-known group of small, diffusible signaling molecules, the acylated homoserine lactones (AHLs), are common to many *Proteobacteria* where they are used to coordinate gene expression, often in a population-density-dependent manner (i.e., quorum sensing [QS]) (8). Canonical AHL-mediated QS consists of a two-component system consisting of a transcriptional regulator (LuxR-type protein) and an AHL synthase (LuxI-type protein) that produces a diffusible ligand. When bound, these protein-ligand complexes can elicit global changes in gene expression (9). Common bacterial traits that are QS regulated include, but are not limited to, bioluminescence, motility, biofilm formation, and antimicrobial production (10–13). Antimicrobials are thought to contribute to microbial interactions principally through growth inhibition of competitors. However, for some microbes, these compounds may themselves function as intermicrobial signals at low concentrations (14). In addition, antimicrobials have been demonstrated to influence biofilm formation in some bacterial species (15, 16). Thus, these two classes of molecules can have overlapping roles and are crucial to competitive and cooperative microbial interactions.

In many environments, diverse microorganisms are enclosed in biofilms where they are in close physical association with one another and encased in a self-produced polymeric matrix (17, 18). The biological and physicochemical properties of biofilms make them ideal systems to study microbial interactions, especially those that are mediated by diffusible small molecules. In addition, biofilms play critical roles in ecosystem functioning, where they mediate transformations key to biogeochemical cycling (19), bioremediation (20), and biofouling (21). In turn, biofilm-associated microorganisms are afforded some degree of protection by the biofilm matrix from external environmental stressors, predators, toxins, and antibiotics (22).

The current understanding of QS-mediated microbial interactions within biofilms is based principally on coculture and natural assemblages (23, 24). While valuable, coculture studies can be limited by the oversimplification of microbial interactions. On the other hand, mesocosm experiments using complex natural communities introduce a wide range of variables to consider when trying to tease apart microbial interactions (25–27). Synthetic intentional communities provide an opportunity to limit community complexity while still allowing for a higher order level of interactions beyond pairwise interactions. In addition, synthetic communities have been used recently in surface colonization and biofilm studies to mimic interactions found in natural ecosystems (28–30). Despite the recent emergence of synthetic multispecies-based biofilm studies, our knowledge of microbial interactions within natural biofilm communities is still relatively incomplete, specifically those cooperative and competitive interactions influenced by small molecules, such as secondary metabolites. A critical aspect to understanding these small-molecule-linked interactions within multispecies biofilm communities is to evaluate the fitness, physiological, and ecological aspects between individual species and a multispecies biofilm consortium.

Due to their predilection to form biofilms on a variety of surfaces and robust secondary metabolite production, members of the heterotrophic *Roseobacteraceae* family of bacteria are ideal model organisms for examining the molecular mechanisms that mediate the cooperative and competitive microbial interactions within biofilm communities (31). These bacteria comprise upward of 20% of microbial communities in coastal marine ecosystems, possess a large genetic repertoire, and demonstrate high metabolic diversity (32, 33). Diverse family members have been found to be primary and aggressive colonizers of a variety of surfaces in coastal oceans (32, 33). The production of secondary metabolites likely contributes to the competitive fitness of *Roseobacteraceae* strains in marine biofilms. For example, genomic evidence suggests the majority of sequenced strains possess at least one QS gene (34). Furthermore, nonribosomal peptide synthases and polyketide synthases (PKSs) are pervasive in marine strains (35).

To evaluate the impact of secondary metabolite production, specifically AHLs and antimicrobials, on microbial interactions in biofilms, we conducted synthetic community experiments using a five-member *Roseobacteraceae* community. The selected strains (Sagittula stellata sp. E-37, *Rhodobacterales* strain Y4I, Roseovarius nubinhibens ISM, *Sulfitobacter* sp. EE-36, and *Citreicella* sp. SE45) are representatives of those found in high abundance in marine environments, where they have been reported to be metabolically active (36, 37). These strains have been studied extensively in their ability to degrade plant-derived aromatic compounds, and each possess genes encoding the protocatechuate pathway, which is responsible for the catabolism of a variety of aromatic compounds, including *p*-coumaric acid (33, 38–40). Additionally, these strains have been used previously in a synthetic community study to evaluate the interactive effects that combinations of labile and recalcitrant substrates have on microbial growth and metabolism (31). One of these strains, *Rhodobacterales* strain Y4I, has been the focus of studies examining the contribution of both quorum sensing and antimicrobials to competitiveness fitness (5, 41, 42). We have access to previously generated Y4I mutants impaired in various aspects of secondary metabolite production and detection (Table 1). Using this synthetic community and substituting different Y4I mutants, we assessed the contribution of secondary metabolites to community dynamics (i.e., community composition and biofilm formation as well as cooperation and competition over time). To achieve this goal, we expanded upon the previous definition of biofilm community cooperation and competition presented by Ren et al. (28). Here, community cooperation is assessed as an increase in biofilm formation compared with the best biofilm producer in monoculture without the loss of total cell viability in the community. Community competition is defined as a decrease in biofilm formation compared with the worst biofilm producer in monoculture or a decrease in viability in the community (28). As secondary metabolite production is linked intrinsically with central metabolism, we also considered the influence of growth medium (i.e., complex versus defined).

**TABLE 1:** *Rhodobacterales* strain Y4I mutant variant secondary metabolite gene expression and indigoidine phenotypes

Strain	Results for:					
	QS2		QS1		Indigoidine biosynthesis^*a*^	
	*pgaR* ^*b*^	*pgaI* ^*b*^	*phaR* ^*b*^	*phaI* ^*b*^	*igiD* ^*b*^	Pigmentation^*b*^^,^^*c*^
*pgaR*::TN5-Km^r^	−	−	−	−	−	−
*phaR*::TN5-Km^r^	+	+	−	−	−	+/−
*phaI*::pKNOCK	+	++	+	−	−	−
*igiD*::TN5-Km^r^	+	++	−	−	−	−

a For indigoidine biosynthesis, expression the biosynthetic igiD gene was assessed as well as the degree of pigmentation when grown on agar, which correlates with indigiodine concentration.

b See reference 42.

c See reference 41.

## RESULTS

### Genomic analysis of QS and secondary metabolites in synthetic community members.

Previous studies have reported that both QS systems and biosynthetic pathways for antimicrobial production (e.g., PKS and nonribosomal polypeptide synthase [NRPS]) are prevalent in *Roseobacteraceae* genomes (5, 39, 43). All five strains used in this study possess at least one QS component. Three of the five strains (E-37, SE45, and Y4I) contain one or more canonical *luxRI* paired QS system(s). Additionally, these strains each harbor an unpaired (i.e., orphan or solo) LuxR homolog. In contrast, two members (ISM and EE-36) possess only orphan LuxR and LuxI homologs (Table 2).

**TABLE 2:** Community members, their *luxRI* homologous genetic loci, and putative AHLs produced by community members

Species and strain	LuxR gene locus	LuxI gene locus
Sagittula stellata E-37	SSE37_11169^*a*^^,^^*c*^	SSE37_11164^*a*^^,^^*c*^
	SSE37_06082^*b*^^,^^*c*^	
*Sulfitobacter* sp. EE-36	EE36_03628^*b*^^,^^*c*^	EE36_01635^*b*^^,^^*c*^
Roseovarius nubinhibens ISM	ISM_09921^*b*^^,^^*c*^	ISM_03755^*b*^^,^^*c*^
	ISM_15650^*b*^^,^^*c*^	
*Citreicella* sp. SE45	CSE45_4055^*a*^^,^^*c*^	CSE45_4054^*a*^^,^^*c*^
	CSE45_1818^*b*^^,^^*c*^	
*Rhodobacterales* strain Y4I	RBY4I_1689^*a*^^,^^*c*^	RBY4I_3631^*a*^^,^^*c*^
	RBY4I_1027^*a*^^,^^*c*^	RBY4I_3464^*a*^^,^^*c*^
	RBY4I_896^*b*^^,^^*c*^	

a Paired QS system.

b Solo *luxR/luxI*.

c See reference 64.

It has been demonstrated previously that Y4I possesses two QS systems (*pgaRI* and *phaRI*) which coordinately regulate the production of the blue pigmented antimicrobial indigoidine, encoded by a nonribosomal polypeptide synthase (NRPS) termed *igiD* (41). NRPS-like genes encode secondary metabolites, such as toxins, antimicrobials, and pigments (reviewed in reference 44). Two other community members have been reported to possess NRPS-like genes, namely, E-37 and ISM (38), but neither these genes nor their products have been characterized. Both SSE37_17955 and ISM_16730 share more than 90% sequence identity to luciferase-like monooxygenase (LLM) class flavin-dependent oxidoreductases in more closely related organisms (i.e., organisms in their respective genera) and are distinct from *igiD* in Y4I. ISM produces a dark brown-orange pigment, while E-37 does not produce pigmentation (45, 46). The gene(s) responsible for pigmentation in ISM have yet to be identified. Whether the NRPS genes found in E-37 and ISM confer antimicrobial properties is currently unknown.

Pairwise comparisons between synthetic community member LuxI homologs to PgaI and PhaI in Y4I revealed that E-37 and SE45 possess more than 50% amino acid identity to PgaI. Both EE-36 and ISM shared more than 25% amino acid identity with PhaI in Y4I (data not shown). The high protein similarity in AHL synthases between community members suggests the synthesis of AHLs with similar structures. Here, we present preliminary evidence that community members likely produce AHLs with overlapping masses corresponding to C5-HSL, C10-HSL, and C12-HSL (see Fig. S1 in the supplemental material).

### Secondary metabolites may underlie interactions between synthetic biofilm members.

In order to determine whether secondary metabolite production among individual community members could inhibit the growth of community residents, we performed a pairwise assessment of interactions between the five synthetic community members using a fully factorial growth inhibition assay. Only one strain, *Rhodobacterales* strain Y4I, was able to inhibit the growth of any of the other strains (2 of 4) (Table 3). Using mutants that are either abolished in indigoidine pigment production (*igiD*::Tn5-Km^r^) or hyper-pigmented (*clpA*::Tn5-Km^r^), the inhibition of strains EE-36 and E-37 was strictly correlated with indigoidine production capability as determined previously (5, 42) (Table 3), indicating this compound could be an important community determinant in this synthetic community. Previous studies demonstrate the competitive nature of Y4I in community members in both coculture and synthetic community studies, likely resulting from indigoidine production (31, 42). Given that competition and surface attachment have been linked to both QS systems and indigoidine, we used Y4I strains harboring disruptions in these pathways to independently assess the impact of secondary metabolites on biofilm community composition and dynamics.

**TABLE 3:** Factorial growth inhibition of synthetic community members

Lawn	Results^*a*^ by inhibitor organism						
	Y4I	*clpA*::Tn5-Km^r^^*b*^	*igiD*::Tn5-Km^r^^*c*^	E-37	EE-36	ISM	SE45
Y4I	X	X	X	−	−	−	−
E-37	+	+	−	X	−	−	−
EE-36	+	+	−	−	X	−	−
ISM	+/−	−	−	−	−	X	−
SE45	−	−	−	−	−	−	X

a +, growth inhibition; +/−, inconsistent growth inhibition; −, no growth inhibition; X, not tested.

b Hyperpigmented Y4I variant.

c Indigoidine null Y4I variant.

### Y4I is an aggressive surface colonizer compared with other community members.

**FIG 1:**
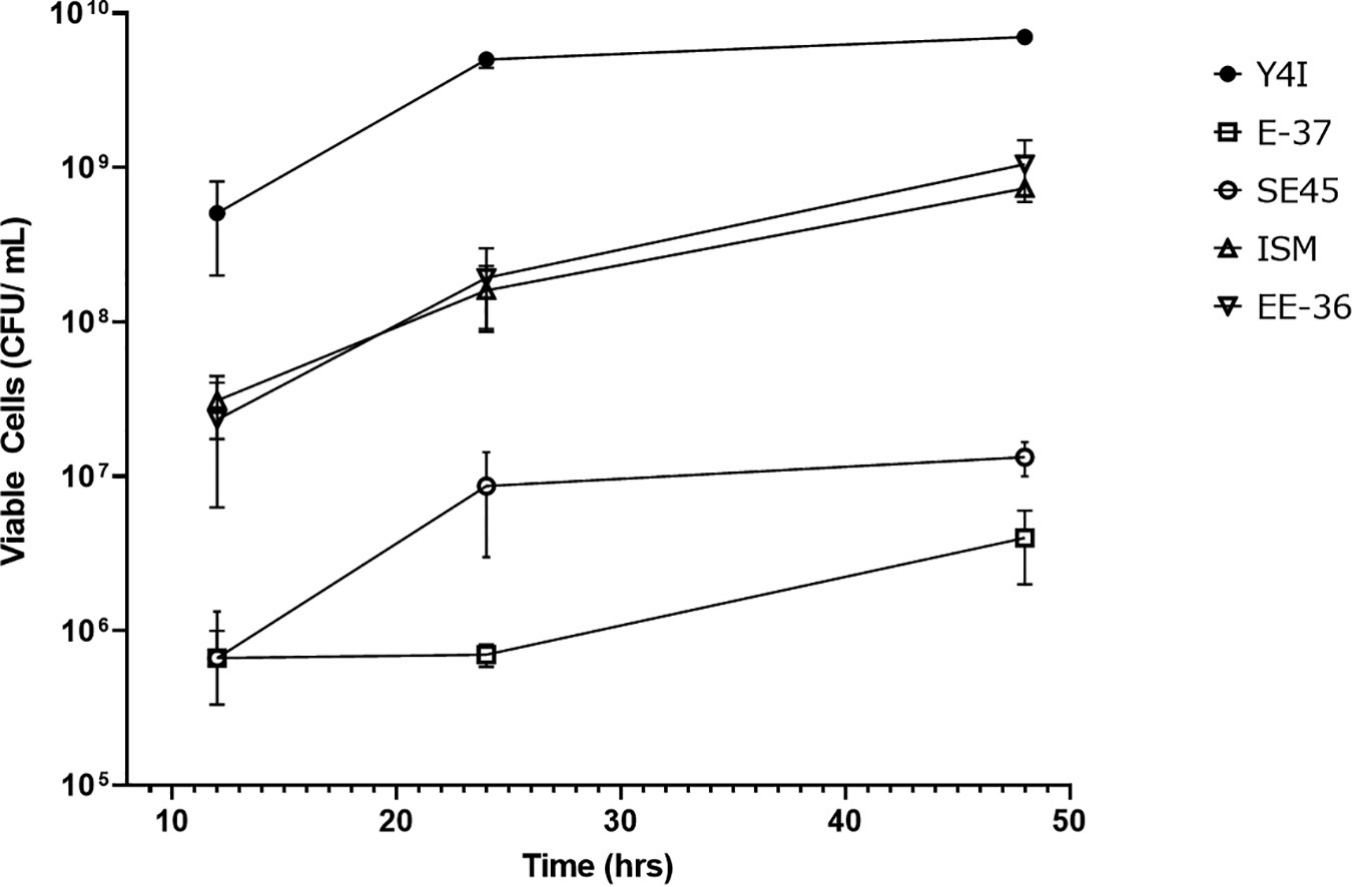
Surface colonization of *Roseobacter* strains to glass beads in a complex medium over 48 h. Viable cell abundance is shown of five *Roseobacter* strains: Y4I (closed circles), E-37 (open squares), SE45 (open circles), ISM (open upward triangles), and EE-36 (open downward triangles). Initial seeding density from liquid culture tubes was ~10^8^ cells mL^−1^. Each data point represents the average of three biological replicates. Error bars represent the standard error of the mean. Some error bars are not visible due to low variance between replicates. All cultures were statistically significant from Y4I at 24 h and 48 h, except EE36 which was only significantly different at 24 h (*P* < 0.05). Y4I variants (not shown) were not significantly different from WT Y4I (Fig. S2). Two-way ANOVA for all strains used in this assay can be found in Table S3.

We assessed the surface colonization of the five individual synthetic community members as well as the Y4I QS and indigoidine variants in monoculture (Fig. 1) at 12, 24, and 48 h postinoculation. Twelve hours after inoculation, the number of viable Y4I cells colonizing the glass beads was at least an order of magnitude higher than that of all other strains and remained significantly greater than all community members at 24 h and 48 h (*P < *0.05), with the exception of EE-36 at the final time point (Fig. 1). Attachment rate and viable cell abundance among Y4I variants were comparable (see Fig. S2 in the supplemental material).

### Secondary metabolites influence biofilm composition.

We next assessed whether QS or antimicrobial production influenced microbial biofilm community structure (composition) and dynamics (cooperation or competition) in the five-member synthetic *Roseobacteraceae* community. Each synthetic community contained one of the following Y4I variants: wild type (WT), *pgaR*::Tn5-Km^r^, *phaR*::Tn5-Km^r^, *phaI*::pKNOCK, and *igiD*::Tn5-Km^r^ (Table 1). Both the *pgaR* and *phaR* variants are unable to sense their cognate AHLs, C8-HSL and 3OHC12:1-HSL, respectively. Additionally, the *pgaR* mutant is unable to produce the indigoidine, while the *phaR* variant expresses delayed and reduced amounts of indigoidine (41). To eliminate this leaky phenotype, we previously generated a mutant in the corresponding AHL synthase gene, *phaI*. This mutant is unable to produce 3OHC12:1-HSL, but transcriptional regulators PhaR and PgaR remain functional. Upon exogenous addition of AHLs, a partial restoration of indigoidine production was restored only in the PhaR/I system with the addition of C8-HSL, alone or in combination with 3OHC12:1-HSL. Thus, the *phaI* variant is able to sense AHLs corresponding to the *phaRI* and *pgaRI* QS systems (42). The indigoidine biosynthesis mutant, *igiD*::Tn5-Km^r^, is unable to produce indigoidine but expresses WT levels of both QS systems (42). These indigoidine phenotypes were evident even within synthetic communities Thus, we could quantitatively assess the discrete and compounded impact of QS systems and antimicrobial production.

**FIG 2:**
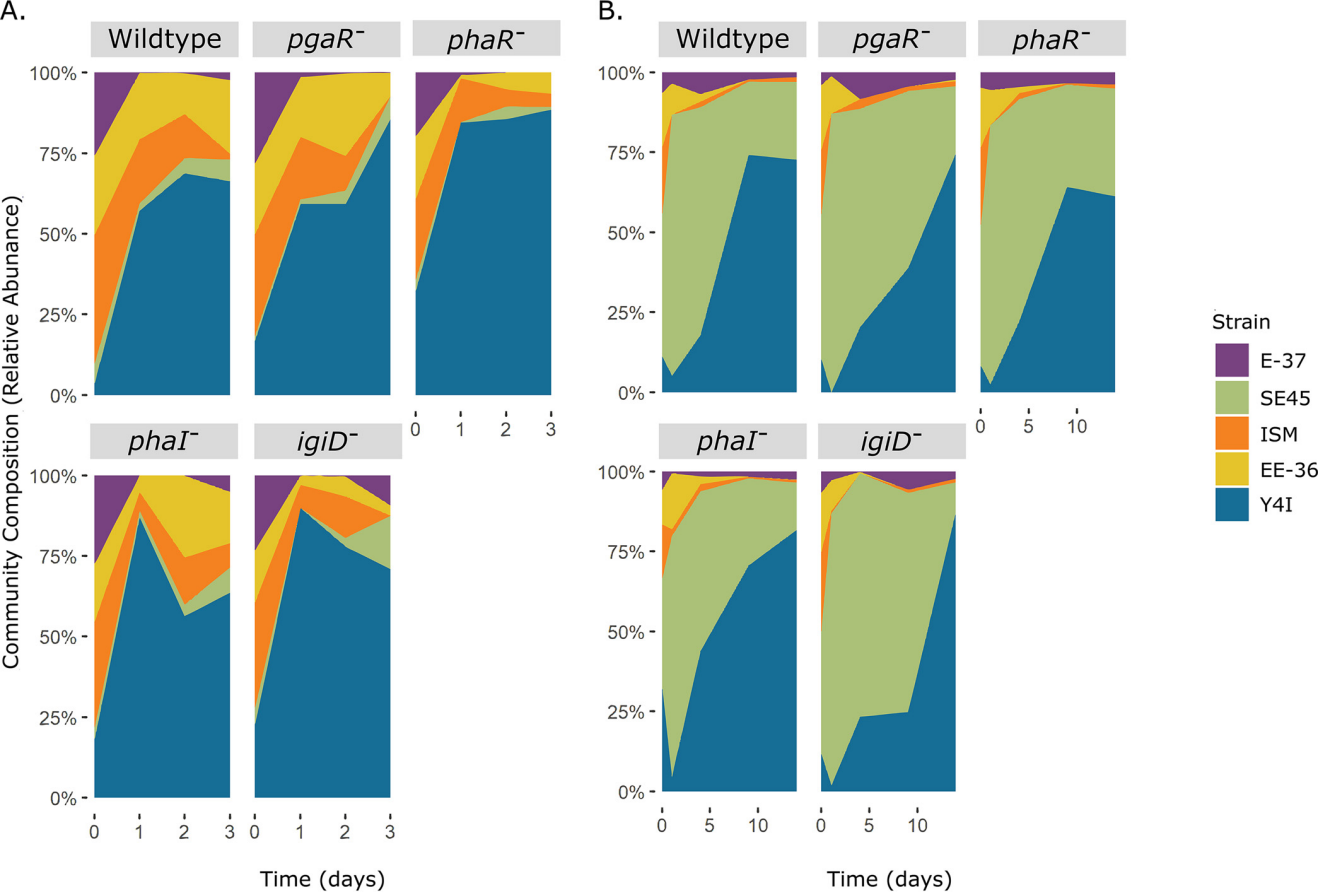
Relative abundance of biofilm community members over time in a complex (A) and defined (B) medium. Each synthetic community as follows is designated by the Y4I variant included (gray boxes): wild-type Y4I, *pgaR*::Tn5-Km^r^ (*pgaR^−^*), *phaR*::Tn5-Km^r^ (*phaR^−^*), *phaI*::pKNOCK (*phaI^−^*), and *igiD*::Tn5-Km^r^ (*igiD^−^*). Relative abundance was calculated from the mean CFU mL^−1^ count of at least six replicates for each strain within each mix and the total CFU mL^−1^ for each mix. Individual strains are color coded according to the key.

Viable cellular abundances for each synthetic community were assessed, and the relative abundance of each community member over time (days) was determined. Across all communities, the relative abundance of each strain remained moderately stable. However, composite community viable cell abundance varied over time according to the presence of specific Y4I variants within synthetic communities. For example, synthetic communities harboring either wild-type Y4I or the *phaR* variant stabilized after day 1, whereas total viable cells in synthetic communities containing an indigoidine null mutant decreased in viability on day 2. In contrast, communities possessing the *pgaR* mutant increased continually in viability over time. Synthetic communities harboring the *phaI* variant decreased initially in viability but rebounded on day 3. Despite the modality of community dynamics within each synthetic community, biofilm communities within the complex medium were characterized by an overwhelming dominance of Y4I regardless of the variant, comprising 57% to 89% of the community (Fig. 2A).

We used a defined medium containing *p-*coumaric acid as the sole carbon source to (i) decrease the competitive advantage of Y4I and (ii) determine if the carbon source affected community interactions. When providing a growth disadvantage using this lignin-related phenolic compound, all Y4I variants displayed an initial decrease in viability in synthetic communities (Fig. 2B). This result is consistent with previous findings and is posited to be a result of toxicity due to *p-*coumaric acid, a weak acid (31). In fact, Y4I variants demonstrated a loss of viability compared with no carbon controls in liquid monocultures when grown on *p-*coumaric acid (see Fig. S3 in the supplemental material). However, when grown in the synthetic communities, the number of viable cells of each Y4I variant increased in the presence of *p-*coumaric acid, which is suggestive of a detoxification provided by other community members. This increase in viable cells was comparable to no-carbon controls (see Fig. S4 in the supplemental material). Despite an initial lag relative to complex medium experiments, all Y4I variants dominated their synthetic communities by the end of the experiment under the defined medium condition (Fig. 2). All synthetic communities grew on *p*-coumaric acid and displayed similar growth dynamics to those observed in complex medium (Fig. 2). Communities harboring wild-type Y4I and the *phaR* variant stabilized on day 9, while the synthetic community possessing the *pgaR* mutant continued to increase in viability. At day 14, communities containing the *phaI* variant appeared to stabilize. From day 1 to day 14, the dominant community member in defined medium biofilms switched from SE45 (75% to 87% on day 1) to Y4I (61% to 87% on day 14), regardless of the Y4I variant included (Fig. 2B).

**FIG 3:**
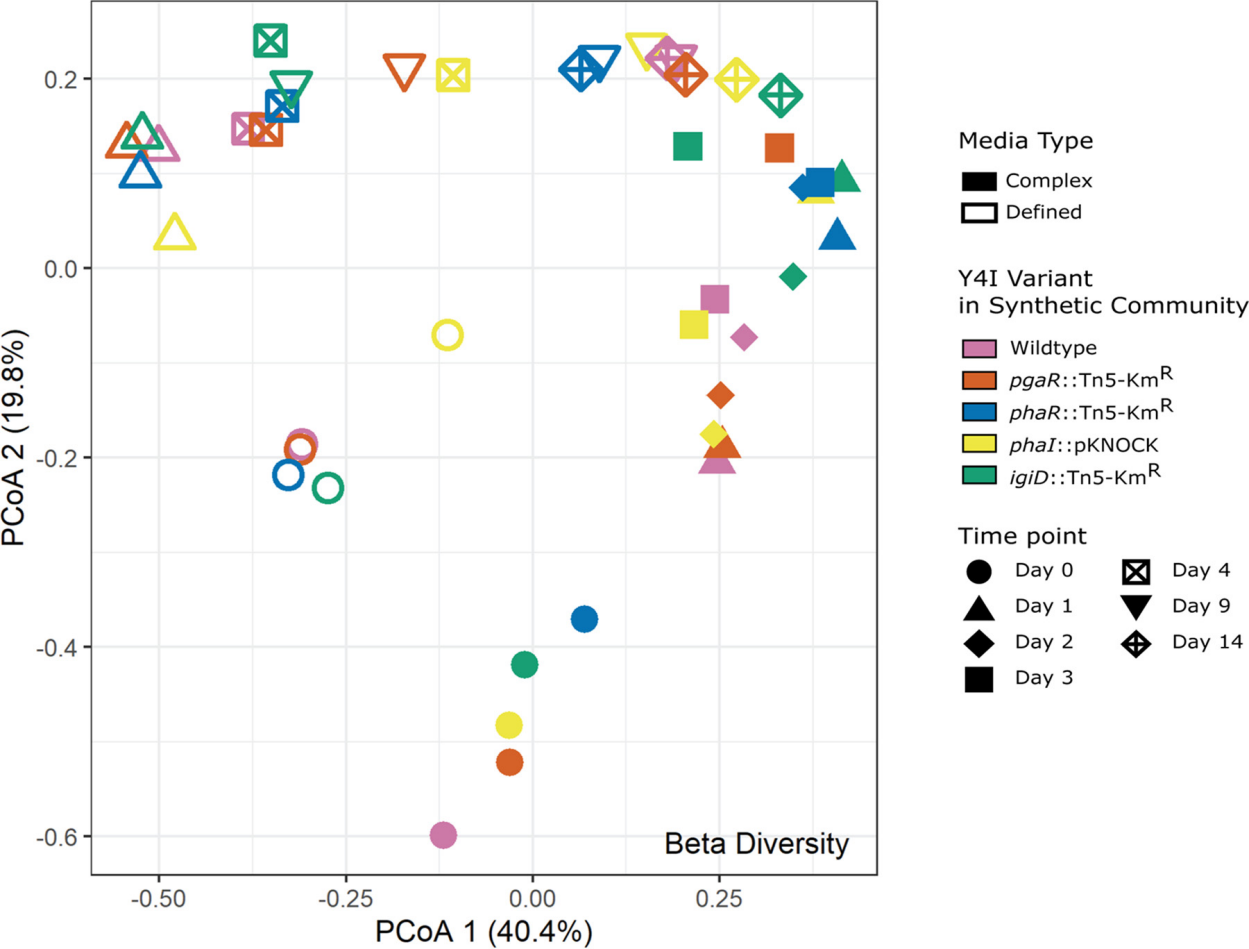
Principal-coordinate analysis (PCoA) plot using the Bray-Curtis dissimilarity index to determine the beta diversity between each carbon source, mixed community, and time point. Medium type is denoted by closed (complex medium) and open shapes (defined medium), synthetic community harboring Y4I variants are differentiated by color, and time point is denoted by shape. At day 0, synthetic communities were not significantly different (*P < *0.05). Statistical analyses are provided in Table S6 to S10.

To investigate the diversity and treatment effects on community structure, we performed alpha and beta diversity analyses using the Shannon index and Bray-Curtis dissimilarity index, respectively. In addition, we used permutational multivariate analysis of variance (PERMANOVA) to measure species richness, species diversity, and significant differences between various communities and growth substrates in each time point (Fig. 3; see Fig. S5 and Table S6 to S10 in the supplemental material). Within each growth substrate experiment, synthetic communities were not significantly different at day 0 (Fig. 3) (*P* < 0.05). From day 0 to day 1 community structures of each synthetic community were dissimilar between growth substrates (i.e., complex versus defined media) but became more similar at later time points (Fig. 3). Across all time points, the structures of each community were significantly different based on growth substrate (*P < *0.05) (Table S6 to S10). Additionally, the structures of synthetic communities on day 1 were significantly different based on not only growth substrate but also which Y4I variant was included in the synthetic community (*P < *0.05) (Table S8). Each time point also was significantly different from other time points between medium types (*P < *0.05) (Table S6 to S10). The alpha diversity of each synthetic community dropped from day 0 to day 1, corresponding to a drop in evenness of the community structure (Fig. S5).

### Secondary metabolites influence biofilm interactions.

**FIG 4:**
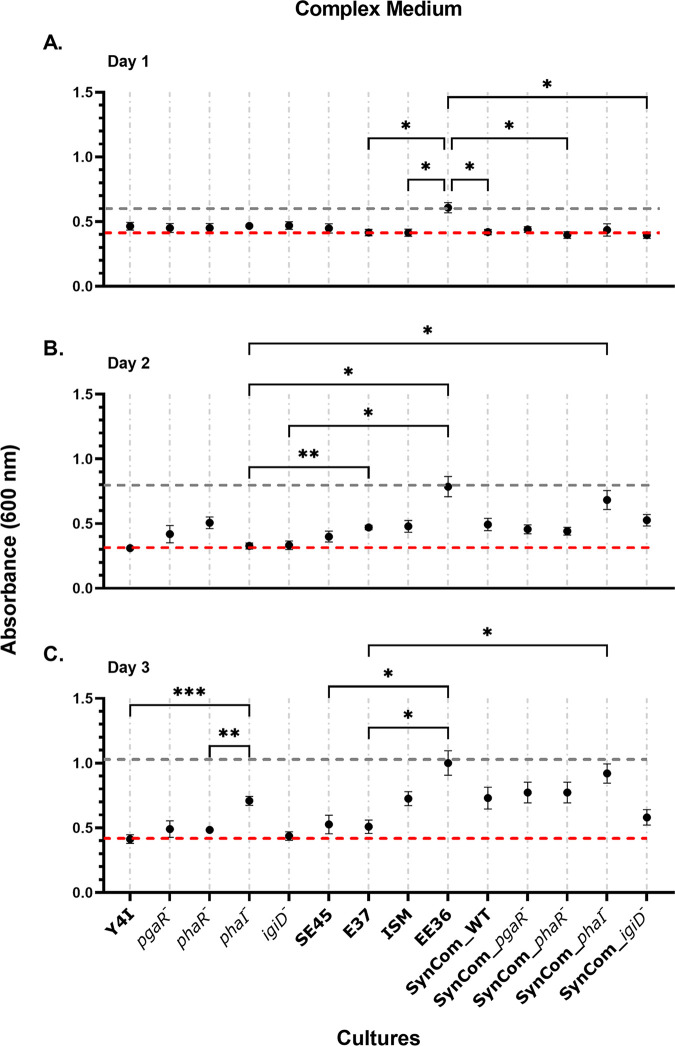
Biofilm production of monoculture community members and five-member synthetic communities in complex medium over time (A to C). Synthetic communities are designated by the Y4I variant in each community (wild-type Y4I, *pgaR*::Tn5-Km^r^ [*pgaR^−^*], *phaR*::Tn5-Km^r^ [*phaR^−^*], *phaI*::pKNOCK [*phaI^−^*], and *igiD*::Tn5-Km^r^ [*igiD^−^*]). Each data point (black dots) was collected by quantifying biomass production using a standard crystal violet assay. Error bars represent the standard error of the mean of three biological replicates and three technical replicates. Some error bars are not visible due to low variance between replicates. The gray dotted line represents biomass production of the best biofilm former in monoculture, and the red dotted line indicates biomass production of the worst biofilm former in monoculture at that time point. Biologically relevant statistical significances are depicted (*, *P < *0.05; **, *P < *0.01; ***, *P < *0.001). For full statistical results see Table S1.

**FIG 5:**
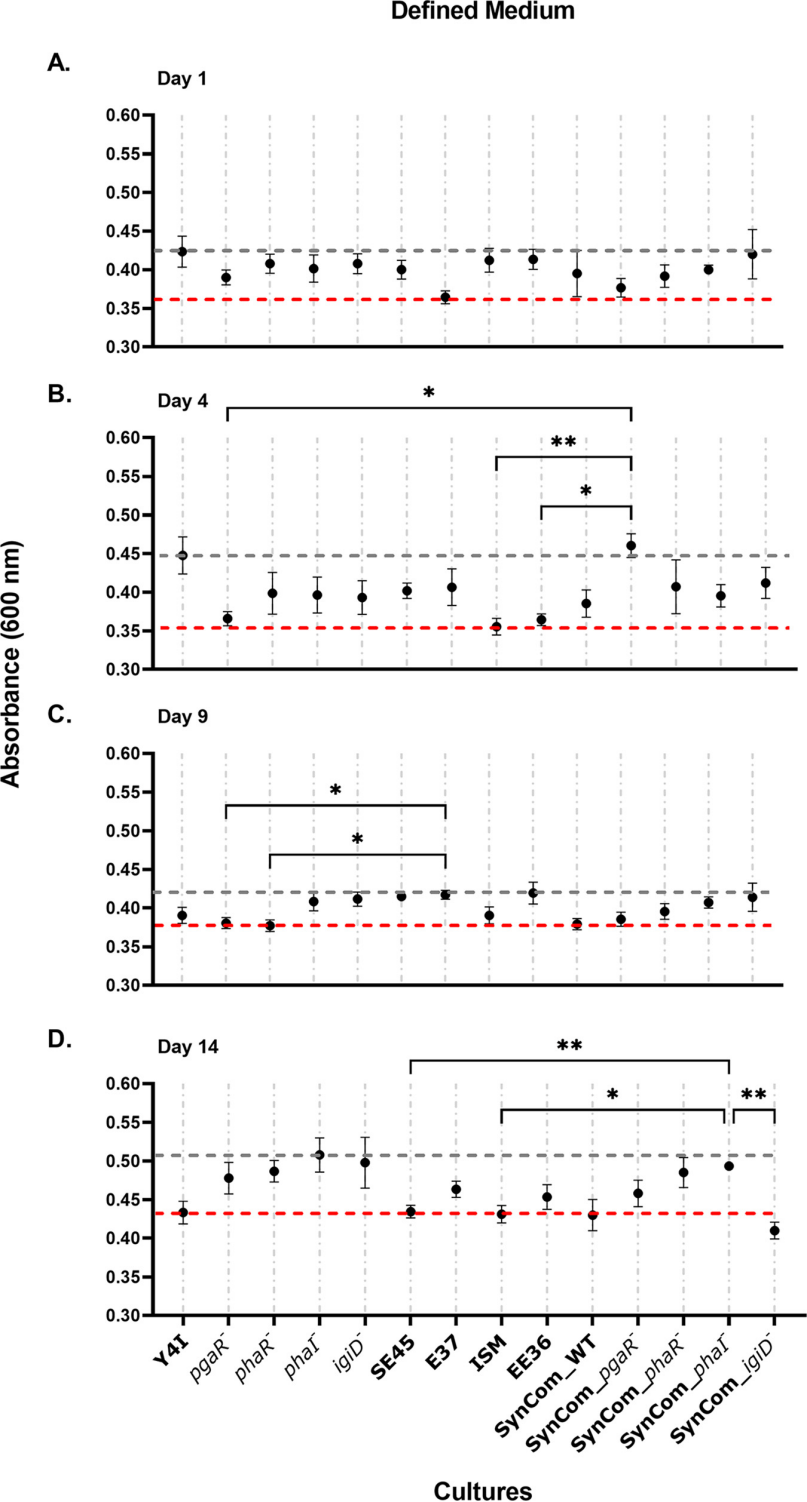
Biofilm production of monoculture community members and five-member synthetic communities in defined medium over time (A to D). Synthetic communities are designated by the Y4I variant in each [wild-type Y4I, *pgaR*::Tn5-Km^r^ (*pgaR^−^*), *phaR*::Tn5-Km^r^ (*phaR^−^*), *phaI*::pKNOCK (*phaI^−^*), and *igiD*::Tn5-Km^r^ (*igiD^−^*)]. Each data point (black dots) was collected by quantifying biomass production using a standard crystal violet assay for at least six replicates for mixed and monocultures. Error bars represent the standard error of the mean. Some error bars are not visible due to low variance between replicates. The gray dotted line represents biomass production of the best biofilm former in monoculture, and the red dotted line indicates biomass production of the worst biofilm former in monoculture at that time point. Asterisks represent statistical significance between cultures (*, *P < *0.05; **, *P* < 0.01). ANOVA table of significance can be found in Table S2.

Viable cell abundance alone cannot fully explain the interactions within mixed species communities. Thus, we assessed the total biofilm production of our five-member synthetic communities across both growth substrates. To determine interactions within mixed communities, biofilm production was measured in both monocultures and synthetic communities using a crystal violet assay where synthetic communities were compared to the “best” and “worst” biofilm producers in monoculture grown on glass beads (Fig. 4 and 5).

When cultures were grown on a complex medium, biofilm production was initially similar among mono- and mixed cultures (optical density at 600 nm [OD_600_], ~0.5), except for EE-36. In this medium, EE-36 produced the most biofilm during each sampling effort (OD_600_ of 0.6, 0.79, and 1.00 on days 1, 2, and 3, respectively). The worst biofilm producer in monoculture switched from ISM (OD_600_, 0.41) on day 1 to Y4I on days 2 (OD_600_, 0.31), and 3 (OD_600_, 0.41) (Fig. 4A to C). Biofilm production in synthetic communities increased over time, with synthetic communities harboring the *phaI*::pKNOCK mutant showing the greatest increase in biofilm production (from OD_600_ of 0.44 to 0.92), over the course of the experiment (Fig. 4B and C). Biofilm formation in all synthetic communities increased over time compared with that of individual monoculture controls (see Fig. S6 in the supplemental material).

Similar to biofilm production observed on day 1 with the complex medium, biofilm production for all mono- and mixed cultures was comparable, with the exception of E-37, in a defined medium containing a less labile carbon source (*p*-coumaric acid). Initially, E-37 produced the least biofilm in monoculture (OD_600_, 0.36) (Fig. 5A). However, the worst biofilm producer switched between ISM (OD_600_ of 0.35, day 4), and Y4I variants (wild type [OD_600_ of 0.43, day 14] and *phaR*::Tn5-Km^r^ [OD_600_ of 0.38 0, day 4]) at later time points (Fig. 5B to D). Y4I variants produced the most and least biofilm throughout this experiment. On day 1 and day 4, the wild type produced the most biofilm (OD_600_ of 0.42 and 0.45, respectively), and the *phaI* variant produced the most biofilm on day 14 (OD_600_ of 0.51) (Fig. 5A, C, and D). Synthetic communities harboring a *pgaR* variant produced slightly more biofilm (OD_600_ of 0.46) than the best biofilm producer in monoculture on day 4, whereas communities containing the *igiD* variant produced less biofilm (OD600_nm_ = 0.41) than the worst biofilm former in monoculture on day 14 (Fig. 5B and D; Table S2). Synthetic communities harboring Y4I variants produced similar amounts of biofilm compared with their monoculture counterparts, with the following exceptions: at day 4, synthetic communities containing the *pgaR* variant had significantly (*P < *0.05) more biofilm production than its monoculture counterpart (Fig. 5B, see Fig. S7 in the supplemental material). Conversely, the synthetic community, including the *igiD* variant showed a decrease in biofilm formation compared with its monoculture counterpart at day 14 (Fig. 5B, Fig. S7). In general, synthetic communities demonstrated a similar modality in biofilm production compared with their monoculture counterparts as either an increase in biofilm production over time or variations of cyclic increases and decreases in biofilm formation.

**FIG 6:**
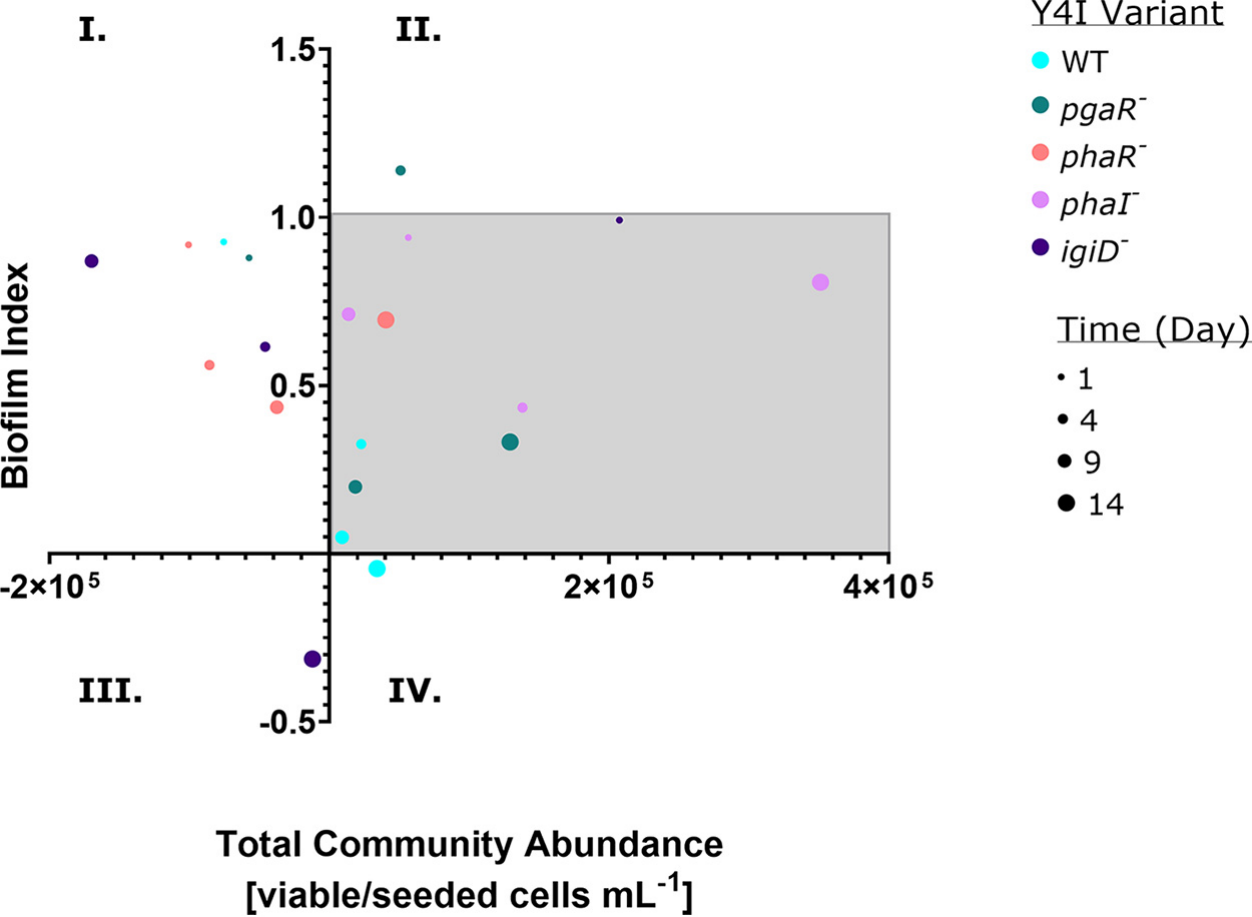
Biofilm production index of synthetic communities harboring Y4I variants in defined medium over time. Y4I variants within communities are color coded according to the legend, and time point varies by the size of dots. Each data point represents the average of at least six replicates. Communities outside gray box indicate the greatest evidence for community cooperation (II) or competition (I, III, and IV).

A Pearson correlation coefficient was used to analyze the growth dynamics of individual species within synthetic communities harboring Y4I variants over time. We focused on investigating interactions observed in minimal media, as the potential influence of secondary metabolite production is more evident within these communities (Fig. 6; see Fig. S8 in the supplemental material). In synthetic communities harboring Y4I variants capable of producing indigoidine, strong negative correlations between Y4I and the community members it has been demonstrated to inhibit, namely, E-37 and EE36 (ρ > −0.75, *n* = 3) (see Table S5 in the supplemental material), were observed. This negative correlation was evident in both medium types. Most community members were positively correlated with each other, excluding Y4I. These positive correlations became more evident with the inclusion of Y4I variants in which secondary metabolite production was disrupted within synthetic communities. For example, ISM is positively correlated with E-37 or EE36 (ρ > 0.65, *n* = 3) (Table S5) within communities harboring Y4I variant with disruptions to their QS systems. These correlations were significant (*P < *0.05) only in synthetic communities harboring the *phaI* variant. ISM is positively correlated only with both E-37 and EE36 when the indigoidine synthase gene is disrupted (ρ > 0.65, *n* = 3) (Table S5).

## DISCUSSION

Here, we assessed the effect of secondary metabolite production and growth substrate on community dynamics and composition in a five-member synthetic community. It has been hypothesized that competitive interactions shape community structure while cooperative interactions stabilize it, thereby affecting the overall function and dynamic of the biofilm (47). In order to visualize these shifts in community dynamics in response to secondary metabolite mutant variants of one strain (Y4I) within a given community, we compared biofilm production and total viable cell abundance over time. We considered an increase in biofilm formation compared with the best biofilm producer in monoculture without the loss of total cell viability in the community to represent cooperation. In contrast, a decrease in biofilm formation compared with the worst biofilm producer in monoculture, or a decrease in viability in the community, represented community competition. We found community cooperation to become increasingly evident in synthetic communities harboring various Y4I secondary metabolite mutants. When secondary metabolite production, such as QS or antimicrobials, are disrupted in Y4I by genetic manipulation, community members have more similar growth dynamics to one another over time, suggesting overall community cooperation. The influence of secondary metabolite production on community dynamics was most evident within the synthetic community where wild-type Y4I was replaced with a QS mutant (*pgaR* variant). This community shifts toward cooperation over time in the presence of either growth substrates, but it is most noticeable in the *p*-coumaric acid growth condition (Fig. 6 and Fig. S8). The *pgaR* variant is impaired in both QS pathways harbored by the parental strain and is unable to produce indigoidine (41, 42). This information may account for the measurable shift toward community cooperation in the synthetic community harboring this mutant when in the presence of a defined growth medium. Given access to more diverse growth substrates (i.e., complex medium), however, fewer competitive interactions were observed (Fig. S8). This finding is analogous to synthetic soil communities in which biofilms evolved to decrease negative interactions and increase cometabolism among community members (28). Further evidence of cooperation is demonstrated by communities containing a different QS mutant, the *phaI* variant. Cooperation within this synthetic community is exhibited by continual increase in biofilm formation and the stability of community member abundance over time (Fig. 2B and 5). The *phaI* variant is also impaired in indigoidine production as well as in the synthesis of its cognate AHL (42). Inclusion of this mutant in synthetic communities allowed us to explore the possibility of QS cross talk between related species.

QS cross talk has been modeled to contribute to competition or cooperation in diverse microorganisms (48). Possible evidence for QS cross talk within these mixed communities is apparent within the synthetic community harboring the *phaI* variant. Three community members (E37, ISM, and EE36) that were always positively correlated in synthetic communities harboring QS mutants and were significantly correlated in the synthetic community containing *phaI* may share overlapping AHLs. Preliminary evidence indicates AHLs with masses corresponding to 3OHC10-HSL (E-37 and ISM), and C5-HSL (E-37, EE36, and ISM) as well as C12-HSL (all examined strains) (Fig. S1). The *phaI* mutant variant is unable to produce 3OHC12:1-HSL but is able to sense both AHLs predominantly produced by Y4I (C8-HSL and 3OHC12:1-HSL) (42). It is plausible the QS systems in Y4I are able to recognize AHLs with similar masses and acyl-chain lengths, including those produced by community members (e.g., C12-HSL) (Fig. S1B). Previous reviews have discussed the promiscuity of AHL ligand binding with noncognate LuxR-like regulatory proteins (49–51). Preliminary cross-talk experiments suggest that the *phaR* and *phaI* mutants produce indigoidine in response to extracellular metabolites produced by other community members. However, the exact metabolite eliciting this response is unknown. Future efforts to determine the molecular structure of these metabolites produced by community members may lead to more robust evidence for QS cross talk. Given that the *phaRI* pathway in Y4I is likely involved in regulating biofilm formation (42), the putative cross talk between strains presented here may contribute to the increased biofilm formation observed in the synthetic community harboring the *phaI* mutant. This type of QS cross talk has been demonstrated in host-microbe models. For example, in plant-associated biofilms, cross talk among rhizosphere bacteria is hypothesized to enhance the biosynthesis of secondary metabolites, compared with nonadherent soil community counterparts, thereby increasing biofilm formation (52).

Community-level competition is evident in synthetic communities harboring either wild-type Y4I or the *igiD* variant (Fig. 6). Given that the *igiD* mutant is unable to inhibit community members in pairwise inhibition assays and yet possesses functional QS systems (Table 1) (see references 41 and 42), interactions beyond those mediated by antimicrobials contribute to competition within these synthetic biofilms. Using a Pearson correlation analysis to investigate how every strain responded to each Y4I variant, we observed more positive correlations and fewer strongly negative correlations between community members within synthetic communities containing the *igiD* variant than communities harboring other Y4I variants. This finding suggests less competition among individual community members with those communities harboring the *igiD*-deficient strain. Overall, WT Y4I displays a strong competitive advantage against other members within synthetic communities. Each member of the synthetic community was negatively correlated with WT Y4I in the Pearson correlation coefficient matrix regardless of growth medium (see Table S4 and S5 in the supplemental material). The findings presented here provide support that secondary metabolite production influences growth dynamics within a biofilm and that Y4I orchestrates community dynamics within these synthetic communities through competitive interactions. These results support previous findings that the majority of species interactions may be competitive in natural environments (53, 54).

In a previous study, primary growth substrates have been shown to impact *Roseobacteraceae* community composition (31). Another study demonstrated that nutrient availability and concentration impact microbial community interactions, specifically growth inhibition (55). Here, we show that synthetic community structures stabilize in a similar manner independent of the growth substrate. Each medium type significantly impacted community structure at day 1 and all subsequent time points. The Y4I variants experienced growth inhibition in liquid monoculture supplemented with *p*-coumaric acid. However, these strains dominate in multispecies communities provided this aromatic acid as a sole carbon substrate. The deviation in growth dynamics between liquid monocultures and multispecies biofilms across all Y4I variants can be explained by the following two possibilities: (i) evidence for cometabolism and (ii) biofilm-mediated defense against harmful substrates. Two strains, namely, SE45 and E-37, have been reported previously to use *p*-coumaric acid as a sole carbon source. An intermediate of *p*-coumaric acid catabolism is *p*-hydroxybenzoic acid (POB), which all community members can degrade through the protocatechuate pathway (31, 39). The degradation of *p*-coumaric acid into exudate metabolites, such as POB, may explain the delayed increase in Y4I variants in these biofilm communities. Alternatively, *p*-coumaric acid is posited to be toxic at high concentrations (>5 mM) (31). It is possible that in a multispecies biofilm, Y4I is protected against the inhibitory action of these metabolites. Previous reports support the protective nature of biofilms against toxic compounds resulting in increased bacterial stress tolerance (56).

The observations presented here provide a direct link between secondary metabolite production in the form of AHLs and antimicrobials on biofilm community structure and dynamics. As AHLs and antimicrobial production are commonly associated with mixed-species biofilms, these findings have broad relevance. Our results support that primary surface colonizers, such as those in *Roseobacteraceae*, influence community interactions through their diverse metabolic capabilities, perhaps irrespective of growth substrate. This conclusion may be particularly relevant in natural systems where growth substrates fluctuate. This work lays the foundation for understanding how small diffusible molecules, including those involved in cell-to-cell communication, influence cooperative and competitive social behaviors within microbial biofilm communities in marine ecosystems.

## MATERIALS AND METHODS

### Synthetic community bacterial strains, growth conditions, and maintenance.

A synthetic, five-member *Roseobacteraceae* community was used to assess the role of secondary metabolites on marine biofilm community structure and formation. Four of the five strains were isolated previously from southeastern United States estuaries or coastal waters (*Rhodobacterales* strain Y4I, Sagittula stellata sp. E-37, *Citreicella* sp. SE45, and *Sulfitobacter* sp. EE-36 [57]). The final strain, Roseovarius nubinhibens ISM, was isolated previously from the Caribbean Sea (46). Previously established Y4I secondary metabolite mutants *igiD*::Tn5-Km^r^, *pgaR*::Tn5-Km^r^, *phaR*::Tn5-Km^r^, and *clpA*::Tn5-Km^r^ were generated using a mini Tn5 transposon system (5, 41). The *phaI*::pKNOCK mutant was generated previously using targeted insertional mutagenesis (42). All Y4I mutant variants carry a chromosomally located kanamycin resistance gene. The growth rates of all mutants in liquid medium have been assessed previously and found to be the same as that of the wild type (5, 42), indicating no gross growth defects.

For routine maintenance, all strains were maintained on yeast extract tryptone and sea salt (YTSS) agar (per L, it included 15 g Instant Ocean [Thermo Fisher Scientific], 15 g agar [Thermo Fisher Scientific], 4 g tryptone, and 2.5 g yeast extract). Y4I strains were passaged routinely in 20% YTSS (per L, it included 15 g Instant Ocean, 0.8 g tryptone, and 0.5 g yeast extract) to reduce flocking in these strains. Each strain was passaged onto basal medium (BM; per L it included 8.7 mM KCl, 8.7 mM CaCl_2_, 43.5 mM MgSO_4_, and 174 mM NaCl with 225 μM K_2_HPO_4_, 13.35 mM NH_4_Cl, 71 mM Tris-HCl [pH 7.5], 68 μM Fe-EDTA, trace metals [7.85 mM nitriloacetic acid, 0.53 mM MnSO_4_ × H_2_O, 0.42 mM CoCl_2_ × 6H_2_O, 0.35 mM ZnSO_4_ × 7H_2_O, 0.038 mM CuSO_4_, 0.11 mM NiCl_2_ × 6H_2_O, 1.16 mM Na_2_SeO_3_, 0.41 mM Na_2_MoO_4_ × 2H_2_O, 0.33 mM Na_2_WO_4_ × 2H_2_O, and 0.25 mM Na_2_SiO_3_ × 9H_2_O], and trace vitamins [0.0020% vitamin H {biotin}, 0.0020% folic acid, 0.0100% pyridoxine-HCl {B6}, 0.0050% riboflavin {B2}, 0.0050% thiamine {B1}, 0.0050% nicotinic acid, 0.0050% pantothenic acid {B5}, 0.0001% cyanocobalamin {B12}, 0.0050% p-aminobenzoic acid]) containing either 10 mM sodium acetate or 2 mM p-hydroxybenzoic acid (POB). All strains were incubated at 30°C in the dark unless otherwise noted.

### Surface attachment to glass beads.

Surface attachment to glass beads was assessed as described previously by Cude et al. (5). Briefly, sterilized 4-mm glass beads (Pyrex, Corning Incorporated Corning, NY) in a 96-well polystyrene plate (Costar, Corning Incorporated Corning, NY) were inoculated in triplicate with the following strains in monoculture: E-37, SE45, EE-36, ISM, wild-type Y4I, *igiD*::Tn5-Km^r^, *pgaR*::Tn5-Km^r^, *phaR*::Tn5-Km^r^, and *phaI*::pKNOCK. Cells were allowed to attach to glass beads for 12 h, 24 h, and 48 h. Following incubation, glass beads were removed from wells, and cells were dislodged from beads as described previously (5, 42). Following extraction from glass beads, cells were serially diluted and plated onto YTSS and incubated at 25°C.

### Synthetic community biofilm experiment design.

All experiments assessed interactive effects of QS by comparing viable cell counts or biofilm biomass in a mixed species community containing different Y4I variants using a complex medium (20% YTSS) or a defined medium containing 2 mM *p*-coumaric acid as the sole carbon source. Five different synthetic community mixes were assembled. Each synthetic community consisted of E-37, SE45, EE-36, ISM, and one of the following Y4I variants: wild-type Y4I, *pgaR*::Tn5-Km^r^, *phaR*::Tn5-Km^r^, or *phaI*::pKNOCK, *igiD*::Tn5-Km^r^.

For complex medium experiments, overnight monoculture liquid YTSS cultures were diluted 10-fold in fresh YTSS and allowed to grow until mid-exponential growth was reached (~3 h). For each culture, cells were harvested by transferring 1-mL culture to a 1.5-mL centrifuge tube and spun at 8,000 rpm for 10 min. The supernatant was withdrawn, and cells were resuspended in fresh YTSS. All cultures were diluted to ~1 × 10^6^ cells mL^−1^. One milliliter of each culture was combined to establish a master mix of ~5 × 10^6^ cells mL^−1^ mixed cultures containing E-37, SE45, EE-36, ISM, and a Y4I variant. Synthetic mixed cultures were then diluted to 1 × 10^6^ cells mL^−1^ and used to inoculate 96-well plates with a final concentration of ~1 × 10^5^ cells mL^−1^. Final dilutions were plated to assess cell abundance of mixed cultures at the time of inoculation. Monoculture controls (~10^5^ cell mL^−1^) were included in both viable cell abundance and total biomass assays. Six replicates of each plate were made to assay viable cell abundance and total biomass on glass beads over time.

Following the inoculation of 96-well plates, each plate was wrapped with dampened cellulose fiber sheets, stored in a sealing high-density polyethylene (HDPE) bag, and incubated at 30°C. At 20 h (day 1), 44 h (day 2), and 68 h (day 3), cells were extracted from beads. Viable cells, community structure, and biofilm biomass were assessed as described below.

For defined medium containing *p*-coumaric acid, the experimental design was similar to the complex medium, with the following exceptions: all cultures were primed by inoculation into BM containing 2 mM POB and were allowed to grow overnight at 30°C. POB cultures (10 mL) were spun down and washed with BM to remove any residual carbon. BM containing *p*-coumaric acid (2 mM) was inoculated with 0.01 to 0.1 mL of culture to achieve an inoculum of 1 × 10^6^ cells mL^−1^. Cells were extracted for assaying at 24 h (day 1), 96 h (day 4), 216 h (day 9), and 336 h (day 14). Eight replicates of each plate were made to assay viable cell abundance and total biomass on glass beads over time.

### (i) Community structure.

Attached cells were extracted as described above for surface attachment assays and plated onto YTSS agar and allowed to incubate at 25°C until colony morphology was discernible (~5 days). The colony morphology of each strain was readily identifiable except for Y4I mutant variants, which lacked the blue pigmentation diagnostic of the wild-type strain (5, 31, 41, 42). Thus, all mixed cultures containing Y4I mutant variants were also plated onto YTSS containing kanamycin (50 μg/mL) as a control to differentiate between Y4I variants from other community members.

### (ii) Biofilm biomass.

The total biomass on biofilm-grown cultures was assessed by performing a crystal violet stain on glass beads as described previously (42). Briefly, liquid was aspirated out of wells. Glass beads were washed with a 1.5% sea salt solution (Instant Ocean) to remove loosely attached cells. Glass beads were extracted using a vacuum apparatus and moved to a clean 96-well plate. Cells attached to glass beads were stained using a 2% crystal violet solution and solubilized with 95% EtOH. Absorbance was read at 600 nm using a microplate reader (Bio Tek Instruments, Inc.; Synergy HT multi-mode microplate reader, SN 270212).

### Inhibition assays.

A factorial growth inhibition assay using synthetic community members was utilized to assess antagonistic interactions among community members. Briefly, liquid cultures of *Roseobacteraceae* strains were grown overnight in YTSS medium at 30°C with shaking. These overnight cultures were diluted 10-fold, and each of the five strains were spread evenly onto individual YTSS agar plates. All strains were grown to mid-exponential phase and then spotted (10 μL) on top of the spread plates. Y4I variants included an indigoidine null mutant (*igiD*::Tn5-Km^r^) and an indigoidine hyper-producing mutant (*clpA*::Tn5-Km^r^). QS mutants *pgaR*::Tn5-Km^r^, *phaR*::Tn5-Km^r^, and *phaI*::pKNOCK were not included in inhibition assays as they have been demonstrated previously to lack inhibitory activity (5, 41, 42). Growth inhibition assays were incubated at 30°C. Zones of clearing surrounding inhibitor organisms were assessed at 24 h postinoculation.

### Data analysis.

Viable cell counts were analyzed and visualized using RStudio v1.4.1106. All viable cell count data were normalized to the mean relative abundance within replicates. Area plots were constructed based on the mean relative abundance of the strains in each mix over their respective time points for each medium type. Beta diversity analysis was performed using the bcdist() function in the ecodist package (58) for each time point for the complex medium and defined medium. Beta dispersion was calculated using the betadisper() function in the vegan package (59). The adonis() function in the vegan package was used to conduct a permutational multivariate analysis of variance (PERMANOVA) on the paired time points and on the aggregated time points for both carbon sources (59). Alpha diversity was calculated within each time point using the Shannon diversity index in the vegan package (60, 61).

Surface attachment assays and total biomass via crystal violet assays were analyzed and visualized using Prism v9.0.0 (GraphPad Software, San Diego, CA; http://www.graphpad.com). Statistical significance was calculated using a two-way analysis of variance (ANOVA). The variability of differences was corrected using Geisser-Greenhouse correction. Multiple comparisons were assessed using Dunnett’s multiple comparisons for surface attachment assays and Tukey’s honestly significant difference (HSD) for crystal violet assays (see Table S1 to S3 in the supplemental material).

In order to assess community dynamics of paired viable cell counts and surface attachment assays, we first generated a biofilm index for synthetic communities relative to the best and worst biofilm production in monoculture (set to 1 and 0, respectively). We then compared this biofilm index to total community abundance to visualize how the microbial community shifts over time in response to the Y4I variant included in the community. To further investigate interactions within the synthetic communities, we then assessed the correlation of each individual strain’s growth dynamics over time via a Pearson correlation coefficient matrix. The Pearson correlation coefficient matrix was performed using the corr() function, subsequent visualization was done using the corrplot() function, and significance values were computed using the rcorr() function (62, 63). To compare the complex and defined medium communities, we selected correlation values that were above 0.6 or below −0.6 for both medium types to further analyze growth dynamics.

### Data availability.

Code and raw data for this study re available online at https://github.com/jwalto12/RoseobacterSynComm. Bacterial strains are available upon request.
