# Supplementary material for: Quorum Sensing and Antimicrobial Production Orchestrate Biofilm Dynamics in Multispecies Bacterial Communities
Source: Microbiol Spectr. 2022 Oct 18;10(6):e02615-22. doi: 10.1128/spectrum.02615-22 (PMC9769649; doi:10.1128/spectrum.02615-22)
Supplement: Supplemental file 1 — Tables S1 to S10, Fig. S1 to S8, and supplemental methods. Download spectrum.02615-22-s0001.pdf, PDF file, 1.1 MB [file spectrum.02615-22-s0001.pdf]

Supplemental Information

For

Armes, Walton, and Buchan 2022

Table S1 Two-way ANOVA Table for crystal violet assay performed for biofilm formation in complex medium (20% YTSS) by Day.

Tukey's multiple comparisons test	**Mean Diff.**	**95.00% CI of diff.**	**Adjusted P Value**	**Mean 1**	**Mean 2**	**SE of diff.**	**N1**	**N2**	**q**	**DF**
**Day 1**										
E37 vs. EE36	-0.1922	-0.3796 to -0.004875	0.042	0.4156	0.6078	0.04684	9	9	5.804	13.72
ISM vs. EE36	-0.1933	-0.3847 to -0.001955	0.0466	0.4144	0.6078	0.04828	9	9	5.664	14.47
EE36 vs. SynComm_Y4I	0.1889	0.007154 to 0.3706	0.0389	0.6078	0.4189	0.04442	9	9	6.013	12.13
EE36 vs. SynComm_*phaR*	0.2111	0.02332 to 0.3989	0.0215	0.6078	0.3967	0.04701	9	9	6.351	13.81
EE36 vs. SynComm_*igiD*	0.2122	0.02550 to 0.3989	0.0199	0.6078	0.3956	0.0466	9	9	6.44	13.58
**Day 2**										
Y4I vs. E37	-0.16	-0.2667 to -0.05333	0.0015	0.31	0.47	0.02713	9	9	8.342	15.22
Y4I vs. EE36	-0.476	-0.8606 to -0.09151	0.0166	0.31	0.786	0.08122	9	7	8.288	6.885
Y4I vs. SynComm_*phaI*	-0.3726	-0.7144 to -0.03087	0.031	0.31	0.6826	0.07614	9	8	6.921	8.181
Y4I vs. SynComm_*igiD*	-0.2164	-0.4262 to -0.006622	0.0415	0.31	0.5264	0.04932	9	8	6.205	10.09

Table S1 Continued

Tukey's multiple comparisons test	**Mean Diff.**	**95.00% CI of diff.**	**Adjusted P Value**	**Mean 1**	**Mean 2**	**SE of diff.**	**N1**	**N2**	**q**	**DF**
phaI::pKNOCK vs. E37	-0.1422	-0.2550 to -0.02941	0.0081	0.3278	0.47	0.02852	9	9	7.052	14.68
phaI::pKNOCK vs. EE36	-0.4583	-0.8422 to -0.07428	0.02	0.3278	0.786	0.0817	9	7	7.932	7.038
phaI::pKNOCK vs. SynComm_*phaI*	-0.3549	-0.6966 to -0.01314	0.0407	0.3278	0.6826	0.07665	9	8	6.547	8.382
igiD::TN5-KmR vs. EE36	-0.4527	-0.8352 to -0.07015	0.0194	0.3333	0.786	0.08487	9	7	7.544	8.06
igiD::TN5-KmR vs. SynComm_*igiD*	-0.3493	-0.6928 to -0.005849	0.0453	0.3333	0.6826	0.08001	9	8	6.174	9.707
SE45 vs. EE36	-0.386	-0.7708 to -0.001222	0.0491	0.4	0.786	0.0886	9	7	6.162	9.255
**Day 3**										
Y4I vs. *phaI::pKNOCK*	-0.2956	-0.4814 to -0.1097	0.0007	0.4133	0.7089	0.04763	9	9	8.776	15.98
Y4I vs. ISM	-0.3122	-0.5643 to -0.06010	0.0101	0.4133	0.7256	0.06276	9	9	7.036	13.38
Y4I vs. EE36	-0.5878	-1.014 to -0.1620	0.0058	0.4133	1.001	0.09978	9	9	8.331	9.952
Y4I vs. SynComm_*phaI*	-0.5067	-0.8480 to -0.1653	0.0028	0.4133	0.92	0.08183	9	9	8.756	11.02

Table S1 Continued

Tukey's multiple comparisons test	Mean Diff.	95.00% CI of diff.	Adjusted P Value	Mean 1	Mean 2	SE of diff.	N1	N2	q	DF
pgaR::TN5-KmR vs. EE36	-0.5108	-0.9654 to -0.05628	0.0216	0.4903	1.001	0.1136	8	9	6.357	13.72
pgaR::TN5-KmR vs. SynComm_*phaI*	-0.4297	-0.8175 to -0.04190	0.0235	0.4903	0.92	0.09826	8	9	6.185	14.87
phaR::TN5-KmR vs. *phaI::pKNOCK*	-0.2244	-0.3820 to -0.06691	0.0036	0.4844	0.7089	0.03831	9	9	8.285	11.82
phaR::TN5-KmR vs. ISM	-0.2411	-0.4819 to -0.0003386	0.0496	0.4844	0.7256	0.05602	9	9	6.087	9.656
phaR::TN5-KmR vs. EE36	-0.5167	-0.9411 to -0.09222	0.0158	0.4844	1.001	0.09568	9	9	7.636	8.536
phaR::TN5-KmR vs. SynComm_*phaI*	-0.4356	-0.7729 to -0.09816	0.0105	0.4844	0.92	0.07679	9	9	8.022	8.847
phaI::pKNOCK vs. *igiD::TN5-KmR*	0.2722	0.08707 to 0.4574	0.0017	0.7089	0.4367	0.04743	9	9	8.117	15.97
igiD::TN5-KmR vs. ISM	-0.2889	-0.5406 to -0.03714	0.0186	0.4367	0.7256	0.06261	9	9	6.525	13.31
igiD::TN5-KmR vs. EE36	-0.5644	-0.9901 to -0.1387	0.0078	0.4367	1.001	0.09969	9	9	8.008	9.92
igiD::TN5-KmR vs. SynComm_*phaI*	-0.4833	-0.8245 to -0.1422	0.0041	0.4367	0.92	0.08172	9	9	8.364	10.97

Table S1 Continued

Tukey's multiple comparisons test	**Mean Diff.**	**95.00% CI of diff.**	**Adjusted P Value**	**Mean 1**	**Mean 2**	**SE of diff.**	**N1**	**N2**	**q**	**DF**
SE45 vs. EE36	-0.4744	-0.9414 to -0.007484	0.0447	0.5267	1.001	0.1184	9	9	5.665	14.96
E37 vs. EE36	-0.4922	-0.9293 to -0.05515	0.0221	0.5089	1.001	0.1073	9	9	6.488	12.4
E37 vs. SynComm_*phaI*	-0.4111	-0.7724 to -0.04982	0.0194	0.5089	0.92	0.09085	9	9	6.4	14.19

Table S2 Two-way ANOVA Table for crystal violet assay performed for biofilm formation in defined medium (p-coumaric acid) by day.

Tukey's multiple comparisons test	**Mean Diff.**	**95.00% CI of diff.**	**Adjusted P Value**	**Mean 1**	**Mean 2**	**SE of diff.**	**N1**	**N2**	**q**	**DF**
**Day 4**										
pgaR::Tn5-Km^R vs. SynComm_*pgaR*	-0.09458	-0.1742 to -0.01500	0.0185	0.3658	0.4603	0.01783	8	6	7.501	8.353
ISM vs. SynComm_*pgaR*	-0.105	-0.1855 to -0.02446	0.0091	0.3553	0.4603	0.01871	9	6	7.937	9.592
EE36 vs. SynComm_*pgaR*	-0.09583	-0.1750 to -0.01665	0.018	0.3645	0.4603	0.0171	8	6	7.926	7.367
**Day 9**										
pgaR::Tn5-Km^R vs. E37	-0.03667	-0.07252 to -0.0008163	0.0427	0.3805	0.4171	0.009063	9	8	5.721	14.67
phaR::Tn5-Km^R vs. E37	-0.04	-0.07816 to -0.001835	0.0364	0.3771	0.4171	0.009449	8	8	5.987	12.98
**Day 14**										
phaR::Tn5-Km^R vs. SynCom_*igiD*	0.07667	0.005380 to 0.1480	0.0304	0.4867	0.41	0.01765	9	6	6.142	13
SE45 vs. SynComm_*phaI*	-0.05889	-0.09902 to -0.01876	0.0024	0.4344	0.4933	0.009904	9	6	8.409	12.75

Table S2 Continued

Tukey's multiple comparisons test	Mean Diff.	95.00% CI of diff.	Adjusted P Value	Mean 1	Mean 2	SE of diff.	N1	N2	q	DF
ISM vs. SynComm_*phaI*	-0.06222	-0.1142 to -0.01020	0.0148	0.4311	0.4933	0.01254	9	6	7.015	11.33
SynComm_*phaI vs.* SynComm_*igiD*	0.08333	0.02655 to 0.1401	0.0057	0.4933	0.41	0.01229	6	6	9.587	7.429

Table S3 Two-way ANOVA Table for surface attachment assays performed over 48 hrs in complex medium (20% YTSS) by hour.

Dunnett's multiple comparisons test	**Mean Diff.**	**95.00% CI of diff.**	**Adjusted P Value**	**Mean 1**	**Mean 2**	**SE of diff.**	**N1**	**N2**	**q**	**DF**
**12 hrs**										
Y4I vs. *pgaR::Tn5-Km*^{R}	-1.6E+09	-3458942107 to 185608773	0.0705	5.07E+08	2.14E+09	4.2E+08	3	3	3.897	3.982
Y4I vs. *phaR::Tn5-Km*^{R}	1.67E+08	-1574661098 to 1907994431	0.9952	5.07E+08	3.4E+08	3.59E+08	3	3	0.4646	3.293
Y4I vs. *phaI::pKNOCK*	2E+08	-1635066898 to 2035066898	0.9812	5.07E+08	3.07E+08	3.38E+08	3	3	0.5915	2.818
Y4I vs. *igiD::Tn5-Km*^{R}	3.57E+08	-1695030280 to 2408363613	0.8026	5.07E+08	1.5E+08	3.19E+08	3	3	1.118	2.322
Y4I vs. *SE45*	5.06E+08	-1808069364 to 2820069364	0.5854	5.07E+08	666667	3.07E+08	3	3	1.649	2
Y4I vs. *E37*	5.06E+08	-1808084299 to 2820084299	0.5854	5.07E+08	666667	3.07E+08	3	3	1.649	2
Y4I vs. *ISM*	4.76E+08	-1830219916 to 2781553250	0.6231	5.07E+08	31000000	3.07E+08	3	3	1.548	2.008
Y4I vs. *EE36*	4.83E+08	-1817896975 to 2784563641	0.6136	5.07E+08	23333333	3.07E+08	3	3	1.573	2.012

Table S3 Continued

Dunnett's multiple comparisons test	**Mean Diff.**	**95.00% CI of diff.**	**Adjusted P Value**	**Mean 1**	**Mean 2**	**SE of diff.**	**N1**	**N2**	**q**	**DF**
**24 hrs**										
Y4I vs. *pgaR::Tn5-Km*^{R}	3.33E+08	-2964783912 to 3631450579	0.9931	5E+09	4.67E+09	6.67E+08	3	3	0.5	3.2
Y4I vs. *phaR::Tn5-Km*^{R}	3.33E+08	-2964783912 to 3631450579	0.9931	5E+09	4.67E+09	6.67E+08	3	3	0.5	3.2
Y4I vs. *phaI::Tn5-Km*^{R}	1.5E+09	-8719300922 to 11719300922	0.9026	5E+09	3.5E+09	1.71E+09	3	3	0.8783	2.508
Y4I vs. *igiD::Tn5-Km*^{R}	2.47E+09	-5367326110 to 10300659444	0.5236	5E+09	2.53E+09	1.42E+09	3	3	1.736	2.762
Y4I vs. SE45	4.99E+09	638530539 to 9344136128	0.0384	5E+09	8666667	5.77E+08	3	3	8.645	2
Y4I vs. E37	5E+09	645733008 to 9352866992	0.0383	5E+09	700000	5.77E+08	3	3	8.659	2
Y4I vs. ISM	4.84E+09	598039490 to 9081960510	0.0385	5E+09	1.6E+08	5.82E+08	3	3	8.322	2.059
Y4I vs. EE36	4.81E+09	696808109 to 8916525224	0.0363	5E+09	1.93E+08	5.87E+08	3	3	8.185	2.138

Table S3 Continued

Dunnett's multiple comparisons test	**Mean Diff.**	**95.00% CI of diff.**	**Adjusted P Value**	**Mean 1**	**Mean 2**	**SE of diff.**	**N1**	**N2**	**q**	**DF**
**48 hrs**										
Y4I vs. *pgaR::Tn5-Km*^{R}	4E+09	-1712506639 to 9712506639	0.1292	7E+09	3E+09	1.15E+09	3	3	3.464	3.2
Y4I vs. *phaR::Tn5-Km*^{R}	4E+09	-353567306 to 8353567306	0.0588	7E+09	3E+09	5.77E+08	3	3	6.928	2
Y4I vs. *phaI::Tn5-Km*^{R}	2.33E+09	-964783912 to 5631450579	0.1259	7E+09	4.67E+09	6.67E+08	3	3	3.5	3.2
Y4I vs. *igiD::Tn5-Km*^{R}	1.33E+09	-6768050708 to 9434717375	0.8893	7E+09	5.67E+09	1.45E+09	3	3	0.9177	2.725
Y4I vs. SE45	6.99E+09	2633363954 to 11339969379	0.0199	7E+09	13333333	5.77E+08	3	3	12.1	2
Y4I vs. E37	7E+09	2642527953 to 11349472047	0.0198	7E+09	4000000	5.77E+08	3	3	12.12	2
Y4I vs. ISM	6.27E+09	2014152884 to 10519180449	0.0231	7E+09	7.33E+08	5.81E+08	3	3	10.78	2.053
Y4I vs. EE36	3.63E+09	-12341969691 to 19608636358	0.6301	7E+09	3.37E+09	2.4E+09	3	3	1.513	2.244

Table S4. Pearson correlation analysis of individual community members within synthetic communities in a complex growth medium (20 % YTSS). Pearson correlation coefficients were calculated for all community members within a given synthetic community harboring different Y4I variants (top row). Cells with same color scheme corresponds to trends shared across growth mediums. Green color scheme denotes shared positive correlations and red color scheme represents shared negative correlations with a threshold of +/- 0.6. Yellow color scheme indicates self-correlations which are always equal to 1. Significance was unable to be calculated for this data.

	**WT**					***pgaR*::TN5-Km^R**					***phaR*::TN5-KmR**				
	**E37**	**EE36**	**ISM**	**SE45**	**Y4I**	**E37**	**EE36**	**ISM**	**SE45**	**Y4I**	**E37**	**EE36**	**ISM**	**SE45**	**Y4I**
**E37**	1.00	0.61	0.84	0.40	-0.98	1.00	0.32	0.83	-0.53	-0.91	1.00	0.94	0.92	0.44	-1.00
**EE36**	0.61	1.00	0.35	0.31	-0.62	0.32	1.00	0.62	-0.66	-0.66	0.94	1.00	0.73	0.55	-0.94
**ISM**	0.84	0.35	1.00	-0.14	-0.92	0.83	0.62	1.00	-0.92	-0.96	0.92	0.73	1.00	0.21	-0.92
**SE45**	0.40	0.31	-0.14	1.00	-0.21	-0.53	-0.66	-0.92	1.00	0.78	0.44	0.55	0.21	1.00	-0.46
**Y4I**	-0.98	-0.62	-0.92	-0.21	1.00	-0.91	-0.66	-0.96	0.78	1.00	-1.00	-0.94	-0.92	-0.46	1.00

Table S4 Continued

	phaI::pKNOCK					*igiD*::TN5-KmR				
	**E37**	**EE36**	**ISM**	**SE45**	**Y4I**	**E37**	**EE36**	**ISM**	**SE45**	**Y4I**
**E37**	1.00	0.15	0.92	-0.03	-0.90		0.85	0.72	0.32	-0.97
**EE36**	0.15	1.00	0.43	0.24	-0.57	0.85	1.00	0.97	-0.15	-0.95
**ISM**	0.92	0.43	1.00	-0.22	-0.96	0.72	0.97	1.00	-0.38	-0.85
**SE45**	-0.03	0.24	-0.22	1.00	-0.05	0.32	-0.15	-0.38	1.00	-0.15
**Y4I**	-0.90	-0.57	-0.96	-0.05	1.00	-0.97	-0.95	-0.85	-0.15	1.00

Table S5. Pearson correlation analysis of individual community members within synthetic communities in a defined growth medium (*p*-coumaric acid). Pearson correlation coefficients were calculated for all community members within a given synthetic community harboring different Y4I variants (top row). Cells with same color scheme corresponds to trends shared across growth mediums. Green color scheme denotes shared positive correlations and red color scheme represents shared negative correlations. with a threshold of +/- 0.6. Yellow color scheme indicates self-correlations which are always equal to 1. Corresponding p-values are in parenthesis

	WT					*pgaR*::TN5-Km[R]					*phaR*::TN5-KmR				
	**E37**	**EE36**	**ISM**	**SE45**	**Y4I**	**E37**	**EE36**	**ISM**	**SE45**	**Y4I**	**E37**	**EE36**	**ISM**	**SE45**	**Y4I**
**E37**	1 (0)	0.56 (0.33)	0.56 (0.32)	0.55 (0.34)	-0.77 (0.13)	1 (0)	-0.35 (0.56)	0.1 (0.87)	0.09 (0.88)	-0.09 (0.88)	1 (0)	0.68 (0.20)	0.22 (0.72)	0.86 (0.06)	-0.95 (0.01)
**EE36**	0.56 (0.33)	1 (0)	0.81 (0.10)	0.38 (0.53)	-0.76 (0.14)	-0.35 (0.56)	1 (0)	0.77 (0.13)	0.17 (0.78)	-0.65 (024)	0.68 (0.20)	1 (0)	0.81 (0.10)	0.28 (0.65)	-0.8 (0.1)
**ISM**	0.56 (0.32)	0.81 (0.10)	1 (0)	-0.11 (0.87)	-0.39 (0.52)	0.1 (0.87)	0.77 (0.13)	1 (0)	-0.27 (0.66)	-0.31 (0.61)	0.22 (0.72)	0.81 (0.10)	1 (0)	-0.21 (0.74)	-0.44 (0.46)
**SE45**	0.55 (0.34)	0.38 (0.53)	-0.11 (0.87)	1 (0)	-0.87 (0.05)	0.09 (0.88)	0.17 (0.78)	-0.27 (0.66)	1 (0)	-0.83 (0.08)	0.86 (0.06)	0.28 (0.65)	-0.21 (0.74)	1 (0)	-0.78 (0.12)
**Y4I**	-0.77 (0.13)	-0.76 (0.14)	-0.39 (0.52)	-0.87 (0.05)	1 (0)	-0.09 (0.88)	-0.65 (0.24)	-0.31 (0.08)	-0.83 (0.08)	1 (0)	-0.95 (0.01)	-0.8 (0.10)	-0.44 (0.46)	-0.78 (0.12)	1 (0)

Table S5 Continued

	phaI::pKNOCK					*igiD*::TN5-KmR				
	**E37**	**EE36**	**ISM**	**SE45**	**Y4I**	**E37**	**EE36**	**ISM**	**SE45**	**Y4I**
**E37**	1 (0)	0.01 (0.99)	0.9 (0.04)	-0.48 (0.42)	0.09 (0.89)	1 (0)	0.57 (0.32)	0.68 (0.21)	-0.19 (0.77)	-0.27 (0.66)
**EE36**	0.01 (0.99)	1 (0)	0.38 (0.52)	0.8 (0.11)	-0.94 (0.02)	0.57 (0.32)	1 (0)	0.87 (0.05)	-0.03 (0.96)	-0.55 (0.34)
**ISM**	0.9 (0.04)	0.38 (0.52)	1 (0)	-0.06 (0.93)	-0.33 (0.58)	0.68 (0.21)	0.87 (0.05)	1 (0)	-0.33 (0.59)	-0.28 (0.64)
**SE45**	-0.48 (0.42)	0.8 (0.11)	-0.06 (0.93)	1 (0)	-0.91 (0.03)	-0.19 (0.77)	-0.03 (0.96)	-0.33 (0.59)	1 (0)	-0.8 (0.10)
**Y4I**	0.09 (0.89)	-0.94 (0.02)	-0.33 (0.58)	-0.91 (0.03)	1 (0)	-0.27 (0.66)	-0.55 (0.34)	-0.28 (0.64)	-0.8 (0.10)	1 (0)

Table S6. PERMANOVA on all mixed communities on either a complex carbon source or a minimal carbon source.

Variable	Df	SumsOfSqs	MeanSqs	F.Model	R2	Pr(>F)
carbon	1	2.4154	2.4154	56.12	0.37274	0.000666
syn_comm	1	0.0427	0.04273	0.993	0.00659	0.389074
carbon: syn_comm	1	0.0381	0.03808	0.885	0.00588	0.41439
Residuals	37	1.5925	0.04304	0.24575		
Total	44	6.4801	1			

Table S7. PERMANOVA on all mixed communities for the Day 0 time point on either a complex or a minimal carbon source.

Variable	Df	SumsOfSqs	MeanSqs	F.Model	R2	Pr(>F)
carbon	1	0.39274	0.39274	33.475	0.804	0.002665
syn_comm	1	0.02636	0.02636	2.247	0.05396	0.154564
carbon:syn_comm	1	-0.00101	-0.00101	-0.086	-0.00207	0.974017
Residuals	6	0.07039	0.01173	0.14411		
Total	9	0.48848	1			

Table S8. PERMANOVA on all mixed communities for the Day 1 time point on either a complex or a minimal carbon source.

Variable	Df	SumsOfSqs	MeanSqs	F.Model	R2	Pr(>F)
carbon	1	1.90778	1.90778	361.01	0.94121	0.001332
syn_comm	1	0.0325	0.0325	6.15	0.01604	0.04064
carbon: syn_comm	1	0.05495	0.05495	10.4	0.02711	0.021319
Residuals	6	0.03171	0.00528	0.01564		
Total	9	2.02694	1			

Table S9. PERMANOVA on all mixed communities for the paired Day 2/Day 9 time point on either a complex or a minimal carbon source.

Variable	Df	SumsOfSqs	MeanSqs	F.Model	R2	Pr(>F)
carbon	1	0.42584	0.42584	12.4807	0.62754	0.001332
syn_comm	1	0.01813	0.01813	0.5314	0.02672	0.536309
carbon: syn_comm	1	0.0299	0.0299	0.8763	0.04406	0.41439
Residuals	6	0.20472	0.03412	0.30168		
Total	9	0.67859	1			

Table S10. PERMANOVA on all mixed communities for the paired Day 3/Day 14 time point on either a complex or a minimal carbon source.

Variable	Df	SumsOfSqs	MeanSqs	F.Model	R2	Pr(>F)
carbon	1	0.057908	0.057908	3.7557	0.32038	0.03464
syn_comm	1	0.00662	0.00662	0.4293	0.03662	0.74684
carbon: syn_comm	1	0.023706	0.023706	1.5374	0.13115	0.26249
Residuals	6	0.092513	0.015419	0.51184		
Total	9	0.180746	1			

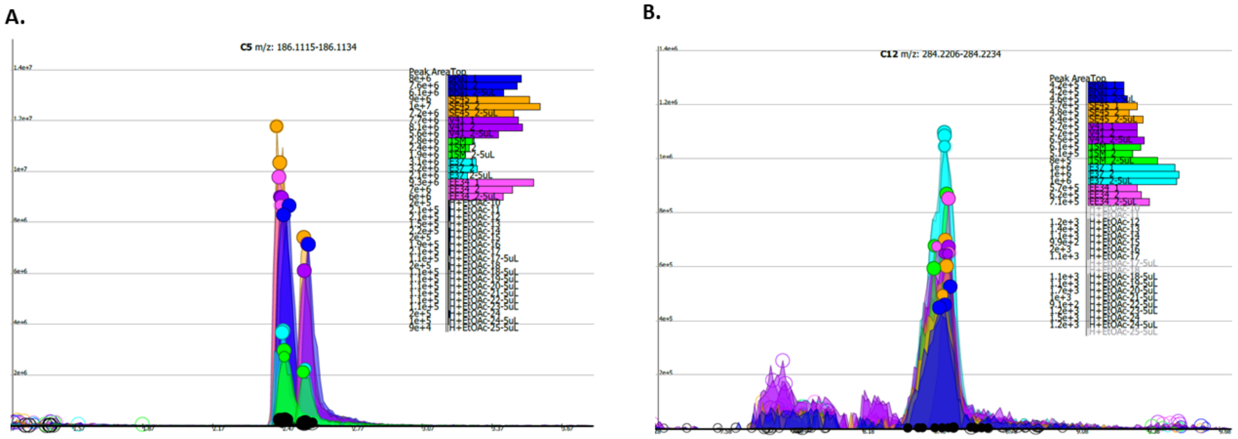

Figure S1. AHL mass peaks (m/z) as measured by HPLC-MS/MS in community members: SE45 (orange), E-37 (cyan), ISM (green), and EE-36 (pink). Y4I variants included in this analysis were WT (purple) and *phaI* mutant (dark blue) as AHL detection for other mutants have been previously published (1).

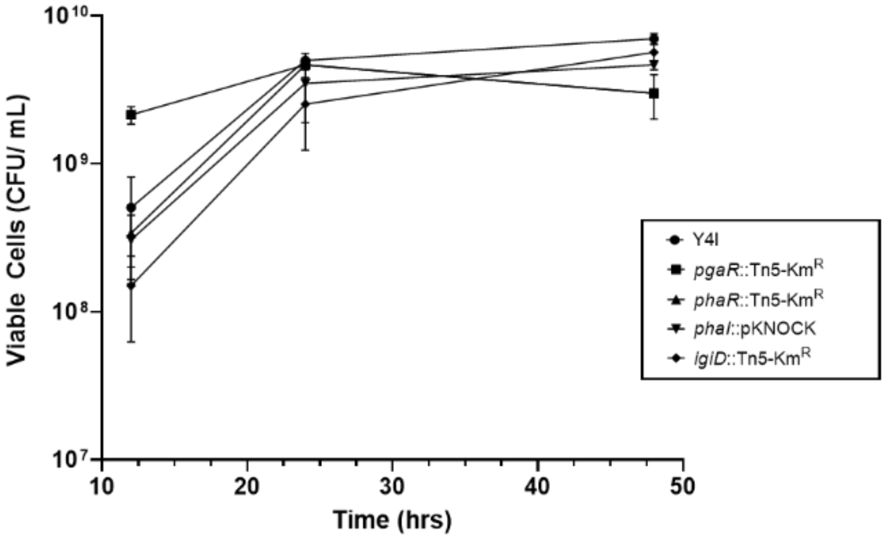

Figure S2. Surface colonization of Y4I variants as they colonize a glass bead in complex medium over time. Viable cell abundance of wildtype Y4I (circles), *pgaR*::Tn5-Km^R (squares), *phaR*::Tn5-Km^R (upward triangles), *phaI*::pKNOCK (downward triangles), and *igiD*::Tn5-Km^R (diamonds) was assessed. Each data point represents the average of three biological replicates. Error bars represent the standard error from the mean.

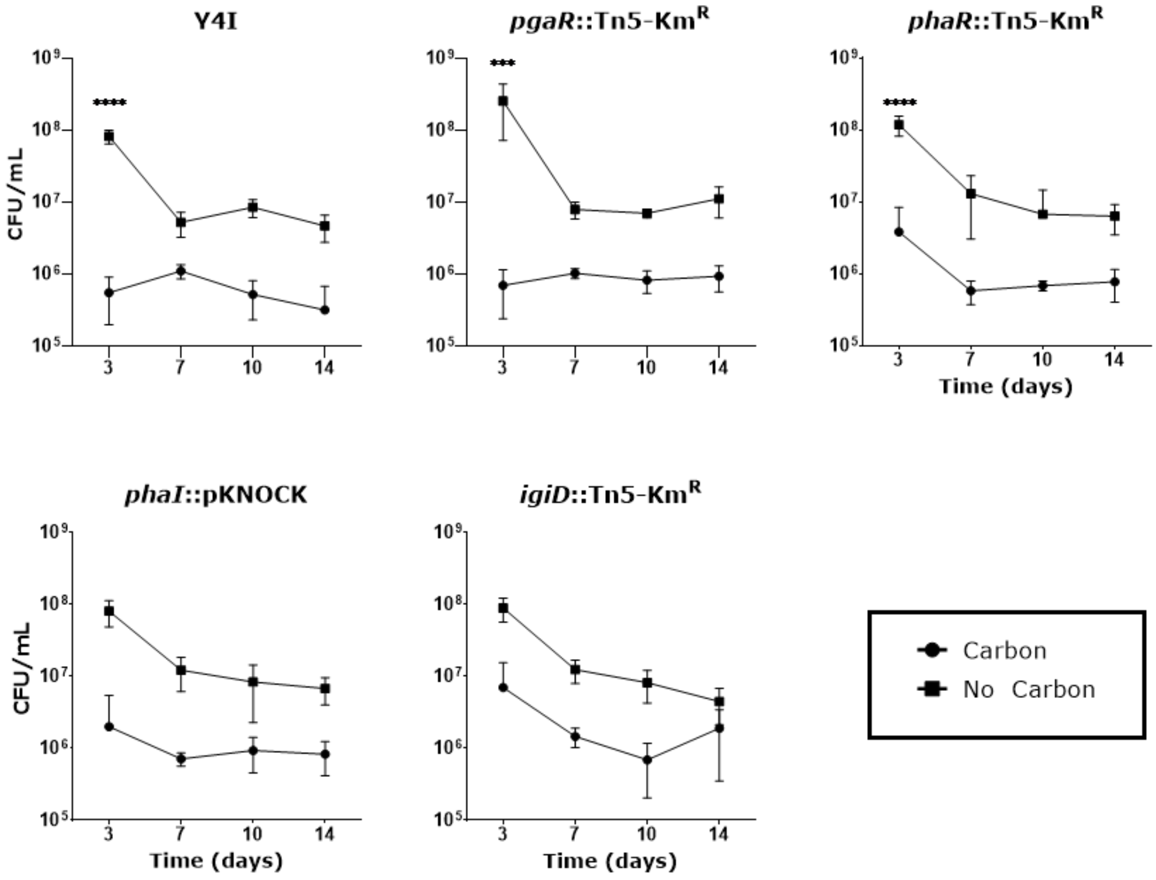

Figure S3. Y4I variants in liquid cultures in the presence of carbon (2 mM *p*-coumaric acid - circles) and no carbon (squares). Y4I variants in liquid cultures in the presence of carbon (2 mM *p*-coumaric acid - circles) and no carbon (squares). Each data point represents the average of three replicates. Error bars represent standard deviation of the mean. Asterisks represent statistical significance [**** ($p < 0.0001$), *** ($p < 0.005$)] using a two-way ANOVA with Sidak's multiple comparisons test as a post hoc test. Inclusion of carbon was significantly different from no carbon control in all analyses ($p < 0.01$).

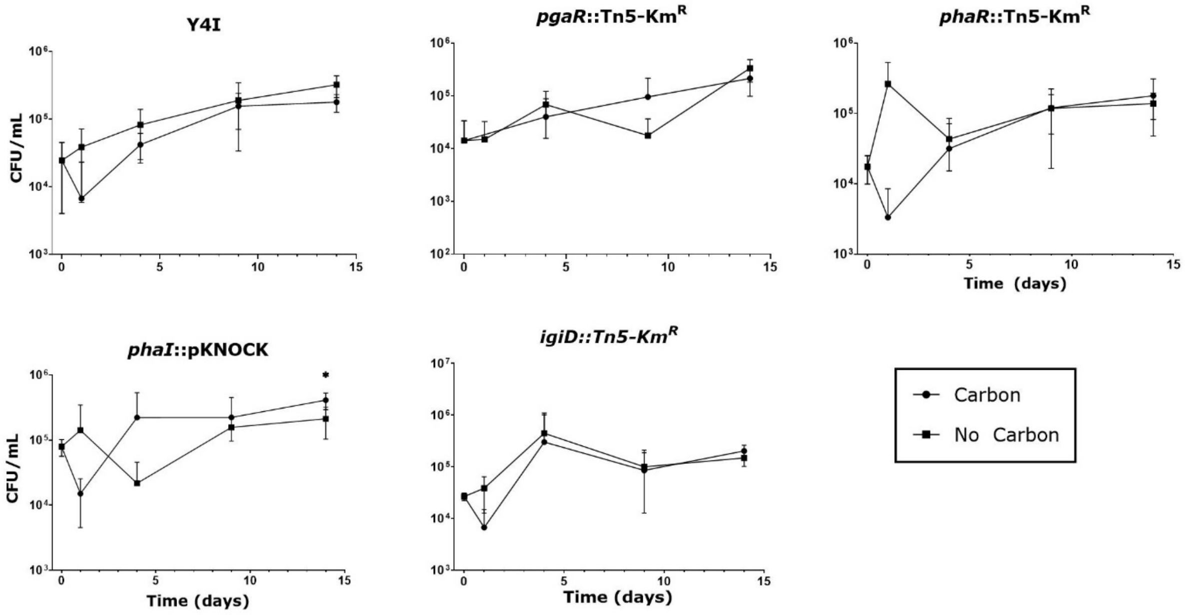

Figure S4. Synthetic mixed biofilm cultures containing Y4I variants (labelled above respective graph) in the presence of carbon (2 mM *p*-coumaric acid - circles) and no carbon (squares). Each data point represents the average of three replicates. Error bars represent standard deviations. Asterisks represent statistical significance ($p < 0.05$) with a two-way ANOVA using Tukey's HSD test as post hoc tests.

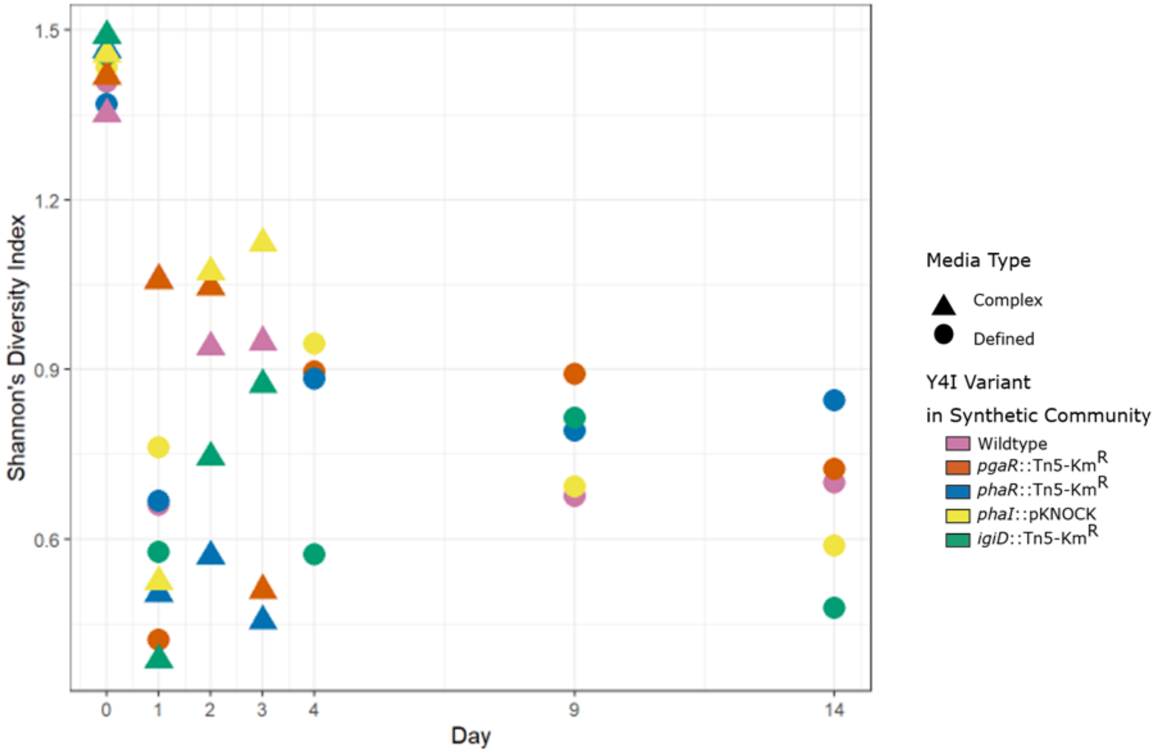

Figure S5. Shannon's Diversity Index for all synthetic communities at each time point based on viable cell counts. Synthetic communities are denoted by color and carbon source (complex or defined/minimal) are designated by triangles or circles, respectively.

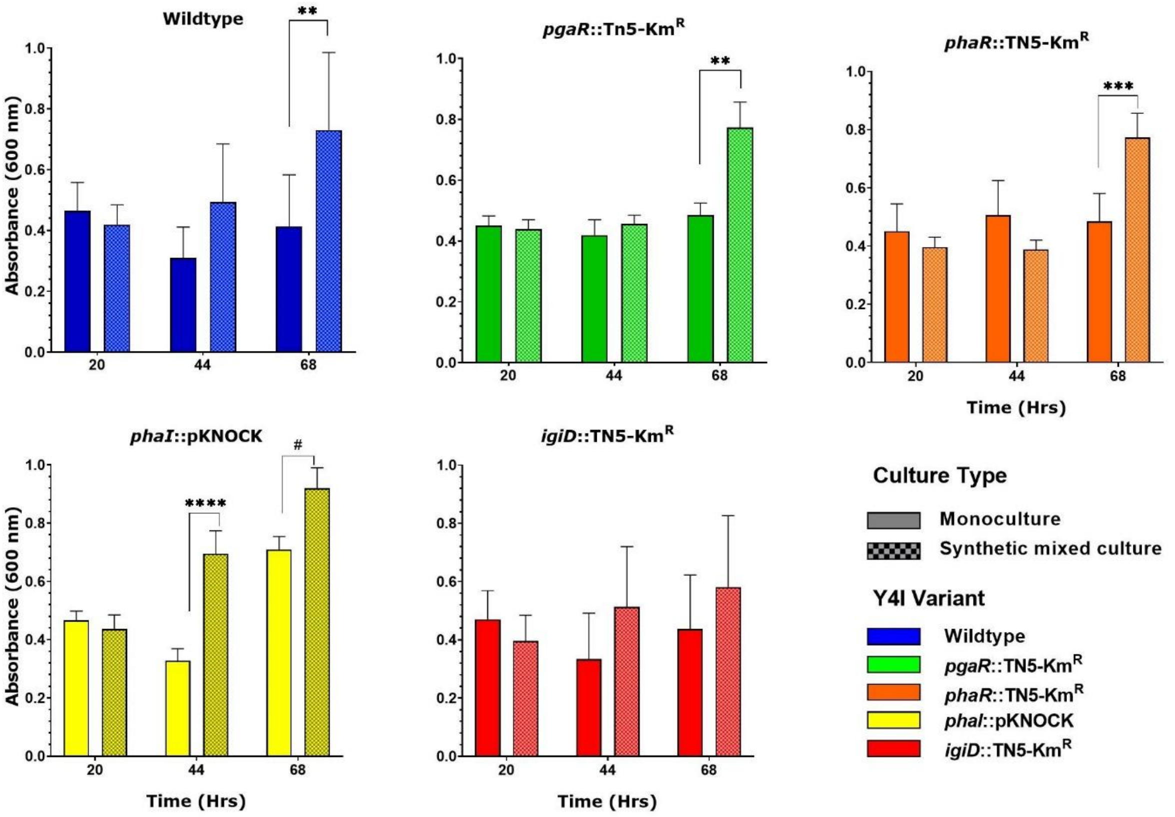

Figure S6. Biofilm production on glass beads from Y4I variants in complex medium over time for monoculture (solid colors) and synthetic mixed cultures (patterned) as determined by crystal violet assay. Data is representative of the mean of three biological and three technical replicates. Error bars represent the standard deviation from the mean. Asterisks denote statistical significance at $p < 0.01$ (**), 0.001 (***), 0.0001 (****). Octothrope represents significance at $p < 0.1$.

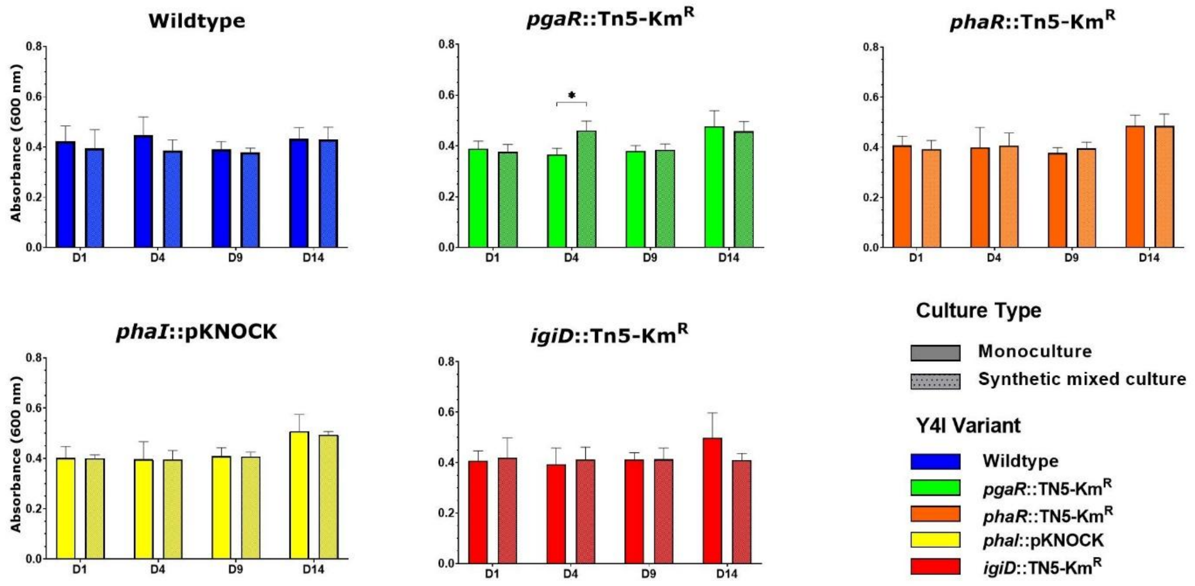

Figure S7. Biofilm production on glass beads from Y4I variants in defined medium over time for monoculture (solid colors) and synthetic mixed cultures (patterned) as determined by crystal violet assay. Data is representative of the mean of three biological and three technical replicates. Error bars represent the standard deviation from the mean. Asterisks denote statistical significance at $p < 0.05$ (*).

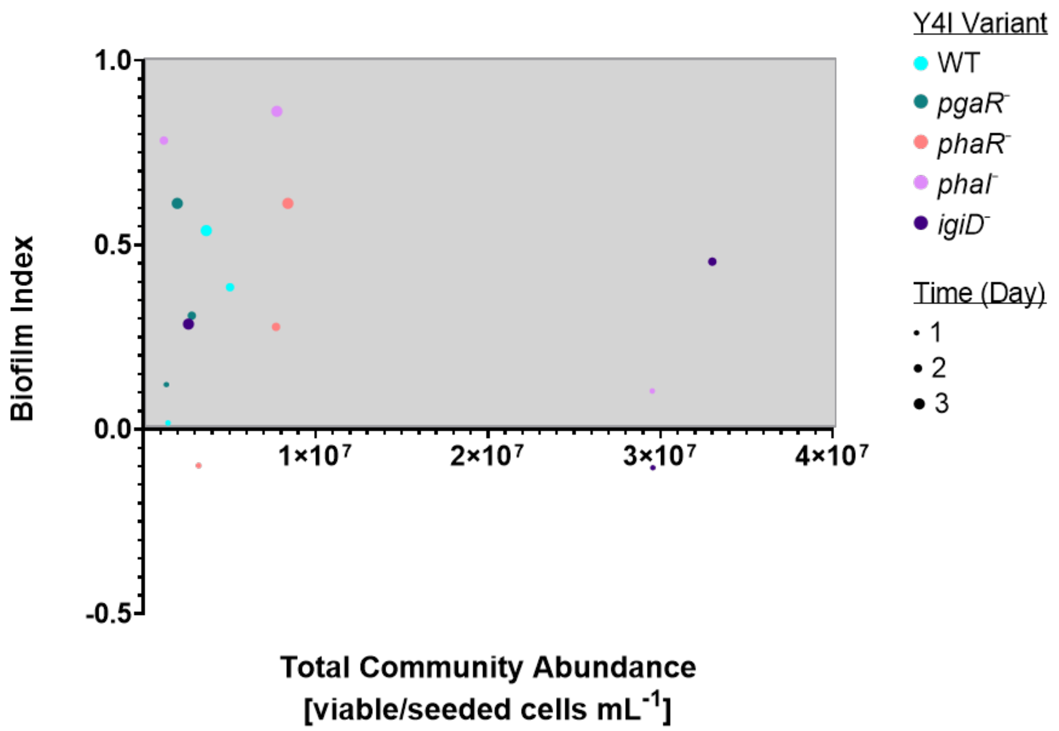

Figure S8. Biofilm production index of synthetic communities harboring Y4I variants in complex medium. Y4I variants within communities are color coded according to the legend and time point at which data was extracted varies by size of dots. Each data point represents the average of 9 replicates. Communities outside of gray box indicate greatest evidence for community cooperation or competition depending on Y4I variant included over time.

Methodology

AHL Extraction and Detection

To assess quorum sensing cross-talk between community members we extracted identified putative AHLs produced by synthetic community members along with two Y4I variants: wildtype Y4I and *phaI*::pKNOCK. AHL extractions on YTSS from *pgaR*::TN5-Km^R and *phaR*::TN5-Km^R mutants have been previously published (2). AHLs were extracted as described in (1). Briefly, mid-exponential phase cultures in triplicate were spot plated onto YTSS agar in a 60 mm petri dish containing a 0.22 uM PES filter and incubated at 30°C for 24 hrs. Filters were aseptically removed and weighed. Cell biomass from filters was extracted and used to estimate microbial abundance. YTSS agar was removed from petri dishes, finely minced, and dissolved in 100 mL ethyl acetate for 3 hrs. Liquid layer was decanted from residual solid layer and vacuum filtered through a silica medium. Extracted AHLs were concentrated in vacuo to produce an oil. This oil was then resuspended in 300 uL acidified ethyl acetate and transferred to an autosampler vial. Samples were stored at -20 °C until analysis. AHLs were detected using high-performance liquid chromatography–tandem mass spectrometry (HPLC-MS/MS) at the Biological and Small Molecule Mass Spectrometry Facility at the University of Tennessee.

References

1. Cude WN, Prevatte CW, Hadden MK, May AL, Smith RT, Swain CL, Campagna SR, Buchan A. 2015. *Phaeobacter sp.* strain Y4I utilizes two separate cell-to-cell communication systems to regulate production of the antimicrobial indigoidine. Appl Environ Microbiol 81:1417–1425.

2. Cude WN, Mooney J, Tavanaei AA, Hadden MK, Frank AM, Gulvik CA, May AL, Buchan A. 2012. Production of the antimicrobial secondary metabolite indigoidine contributes to competitive surface colonization by the marine roseobacter *Phaeobacter sp.* strain Y4I. Appl Environ Microbiol 78:4771–4780.
